# Dual Role of the Spinal Endocannabinoid System in Response to Noxious Stimuli: Antinociceptive Pathways and Neuropathic Pain Mechanisms

**DOI:** 10.3390/ijms262110692

**Published:** 2025-11-03

**Authors:** Raquel Saldaña, Antonio J. Carrascosa, Abraham B. Torregrosa, Francisco Navarrete, María Salud García-Gutiérrez, Jorge Manzanares

**Affiliations:** 1 Servicio de Anestesiologia y Reanimacion, Hospital Universitario 12 de Octubre, Avda. Cordoba s/n, 28041 Madrid, Spain; rasalca@gmail.com (R.S.); acarras3@hotmail.com (A.J.C.); 2 Instituto de Neurociencias, Universidad Miguel Hernández-CSIC, Avda de Ramón y Cajal s/n, 03550 San Juan de Alicante, Spain; a.bailen@umh.es (A.B.T.); fnavarrete@umh.es (F.N.); maria.ggutierrez@umh.es (M.S.G.-G.); 3 Redes de Investigación Cooperativa Orientada a Resultados en Salud (RICORS), Red de Investigación en Atención Primaria de Adicciones (RIAPAd), Instituto de Salud Carlos III, MICINN and FEDER, 28029 Madrid, Spain; 4 Instituto de Investigación Sanitaria y Biomédica de Alicante (ISABIAL), 03010 Alicante, Spain

**Keywords:** endocannabinoid system, neuropathic pain, spinal cord, central sensitization, cannabinoid receptors, antinociception, neuroinflammation

## Abstract

Neuropathic pain is a clinically challenging syndrome that is largely refractory to conventional therapies. It arises from lesions or diseases affecting somatosensory pathways, which trigger extensive neuroplastic and neuroimmune remodeling. Unlike nociceptive pain, which establishes a protective response to tissue injury, neuropathic pain arises from maladaptive signaling within the nervous system. In this context, the spinal endocannabinoid system (ECS) has emerged as a pivotal modulator of nociceptive processing. However, its precise role in neuropathic pain remains debated due to its dual effects. Numerous studies report antinociceptive and neuroprotective effects; however, emerging data indicate that under specific pathological conditions, ECS activation may paradoxically facilitate pain transmission. This review examines spinal ECS context dependence, uncovering its bidirectional antinociceptive and pronociceptive effects in neuropathic pain. By integrating current evidence on cellular, molecular, and pathophysiological mechanisms, we delineate the factors that determine whether ECS modulation inhibits or promotes pain. A comprehensive understanding of these mechanisms is essential for optimizing cannabinoid-based strategies to maximize therapeutic benefits while minimizing adverse outcomes. Finally, we highlight the spinal cord’s centrality as the principal site for the initiation and maintenance of neuropathic pain and advocate for rigorous translational research to clarify the therapeutic potential of spinal ECS-targeted interventions.

## 1. Introduction

**Figure 1:**
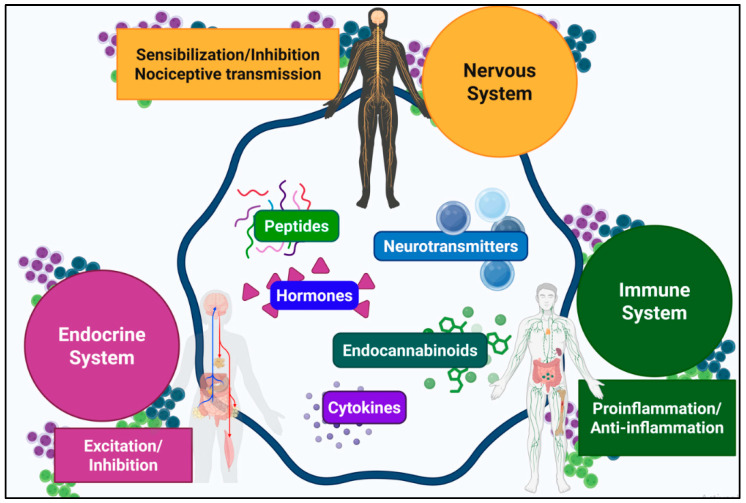
Schematic representation of neuroendocrine–immune communication pathways in response to tissue injury (Note: modified from [1]). Tissue injury activates an integrated defense coordinated by nervous, endocrine, and immune systems, involving bidirectional signaling via autonomic pathways and systemic mediators. Shared molecules mediate context-dependent responses that regulate vascular permeability, immune cell recruitment, and pain thresholds.

Pain perception emerges from the sophisticated interplay of mechanisms operating across multiple levels of the nervous system [1,2]. Noxious stimuli are transduced into electrical signals that travel from peripheral terminals to the dorsal horn (DH) of the spinal cord and subsequently to supraspinal centers, where they are integrated into the conscious experience of pain. As an adaptive defense mechanism, the organism initiates a coordinated biological response involving neural, endocrine, and immune pathways (Figure 1). Throughout the ascending transmission, nociceptive signals are dynamically modulated by both inhibitory and facilitatory mechanisms at spinal and supraspinal levels, leading to either attenuation or amplification of the signal before conscious perception [3,4].

In this context, the DH constitutes the first central relay station for peripheral nociceptive information [5]. Primary afferent fibers are activated by noxious stimuli, generating orthodromic action potentials that propagate from peripheral terminals to the neuronal soma located in the dorsal root ganglion (DRG) [6]. From there, central axonal projections enter the DH, where they synapse onto an intrinsic DH neuron. Spinal projection neurons then convey this processed input to higher centers, contributing to the conscious perception of pain. However, prolonged, intense, or repetitive stimulation can profoundly remodel these sensory-integration circuits, altering the amplitude, duration, and qualitative attributes of nociceptive responses [7].

Neuropathic pain, defined as pain arising from a lesion or disease of the somatosensory system, is one of the most complex and treatment-resistant pain phenotypes encountered in practice [8]. Unlike nociceptive pain, whose peripheral mechanisms are relatively well characterized, neuropathic pain reflects profound neuroplastic and neuroimmune alterations that disrupt standard sensory processing [9]. Understanding the spinal mechanisms underlying this pathological transformation is essential for developing rational therapeutic interventions [10].

## 2. Spinal Cord Physiology and Central Sensitization in Neuropathic Pain

### 2.1. Structural and Functional Organization of the DH

**Figure 2:**
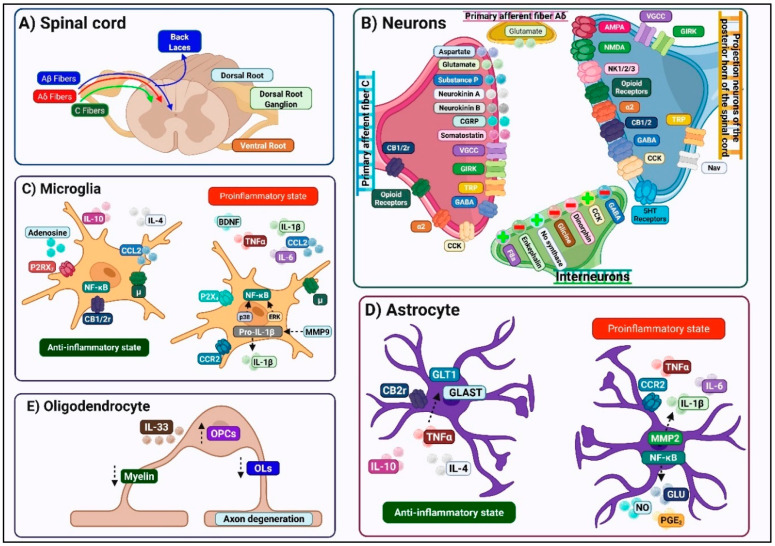
Spinal cord anatomy and cellular elements. Panel (**A**). Spinal cord anatomy. DH (laminae I–VI) integrates sensory afferents from dorsal root ganglia. Aδ and C fibers terminate mainly in laminae I–II, whereas Aβ fibers synapse in laminae III–V, with lamina VI processing proprioception. Lamina X contributes to visceral nociception. Panel (**B**). Neurons. Primary afferent terminals synapse onto second-order projection neurons and local interneurons. Intrinsic nociceptive neurons encode stimulus intensity, while interneurons regulate excitatory drive. Panel (**C**). Microglia. Microglia transition between pro-inflammatory M1 states and anti-inflammatory M2 states. Panel (**D**). Astrocytes. Astrocytes support neuronal metabolism, blood–brain barrier integrity and neurotransmitter clearance. They adopt A1 or A2 phenotypes. Panel (**E**). Oligodendrocytes. These cells provide axonal myelination and metabolic support, and their loss contributes to chronic pain states.

The DH exhibits a highly ordered laminar architecture, first described by Rexed, in which distinct neuronal populations occupy specific rostrocaudal and dorsoventral columns according to their functional roles [11] (Figure 2).

Nociceptive and thermoreceptive primary afferents, comprising thinly myelinated A-delta and unmyelinated C fibers, terminate predominantly within the superficial laminae I and II (substantia gelatinosa), forming synapses onto projection neurons and interneurons specialized for processing noxious and thermal inputs. In contrast, mechanoreceptive A-beta fibers and proprioceptive inputs principally arborize in deeper DH laminae III–V, where they engage circuits mediating tactile discrimination and proprioceptive feedback [12]. This anatomical segregation of sensory modalities underlies the specificity of pain perception under normal conditions. Following synaptic integration in the DH, processed signals ascend via multiple tracts to discrete supraspinal targets. The spinothalamic tract, arising from projection neurons across laminae I–V, conveys both nociceptive and innocuous somatosensory information to thalamic nuclei before relay to cortical regions. In parallel, lamina I neurons also project to the parabrachial nucleus and periaqueductal gray, pathways explicitly implicated in the affective-motivational dimension of pain [12].

Within DH, a convergence of peripheral sensory afferents engages three principal cellular elements: (1) nociceptive projection neurons, which transduce and relay noxious input to supraspinal centers; (2) interneurons, comprising both excitatory and inhibitory subtypes that modulate local circuit function; and (3) glial cells that modulate synaptic function and drive neuroinflammatory processes underlying central sensitization (Figure 2). Nociceptive-projecting neurons in the DH are functionally categorized into nociceptive-specific, wide-dynamic-range, and low-threshold mechanoreceptor-responsive neurons, with molecularly distinct excitatory subtypes (GH+, VGluT3+, protein kinase C (PKC)-gamma+, and NPY1R+) playing key roles in mechanical and cold allodynia [10]. Local interneurons, which represent 99% of DH neurons, further refine nociceptive signaling: inhibitory GABAergic and glycinergic interneurons regulate excitatory drive through axodendritic and axoaxonic synapses, while excitatory glutamatergic interneurons amplify pain transmission and contribute to central sensitization through maladaptive synaptic reorganization [13]. Complementing neuronal activity, glial populations (microglia, astrocytes, and oligodendrocytes) employ profound modulatory effects [14].

Microglia display remarkable phenotypic plasticity, adopting pro-inflammatory (M1) or anti-inflammatory (M2) states that can, respectively, exacerbate or mitigate neuronal hyperexcitability [14,15]. Astrocytes, the most abundant glial cell type, regulate extracellular homeostasis, neurotransmitter clearance, and synaptic integrity, but after injury, they undergo astrogliosis, shifting toward either the neurotoxic A1 or the neuroprotective A2 phenotypes [16]. Oligodendrocytes, critical for axonal myelination and metabolic support, also contribute to pain modulation through activity-dependent myelin remodeling and IL-33–mediated inflammatory signaling [17]. Together, these neuronal and glial interactions establish a finely tuned yet highly plastic spinal network, whose disruption underlies the transition from acute nociception to chronic neuropathic pain [5].

### 2.2. Modulatory Systems Regulating Spinal Nociceptive Transmission

#### 2.2.1. Descending Pain Modulatory Pathways

Descending projections from midbrain, brainstem, and cortical regions exert robust control over spinal nociceptive processing [18]. Under physiological conditions, inhibitory noradrenergic and serotonergic influences predominate over facilitatory serotonergic pathways. However, neuropathic pain states deeply compromise this homeostatic equilibrium, invariably leading to facilitation of spinal excitability and amplified nociceptive transmission [19]. Three primary descending circuits directly influence DH excitability: (1) corticospinal projections from the anterior cingulate cortex that facilitate nociceptive transmission, (2) somatosensory corticospinal fibers that regulate tactile sensitivity and contribute to allodynia, and (3) GABAergic brainstem inputs that paradoxically enhance pain via disinhibition of local inhibitory interneurons.

#### 2.2.2. Spinal Neuroimmune Interactions

**Figure 3:**
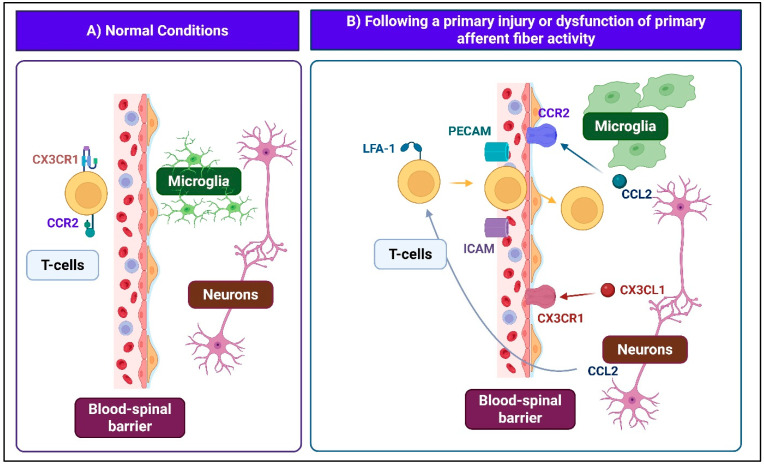
Spinal inflammation. Panel (**A**). Normal conditions. The intact blood–spinal cord barrier and an immunosuppressive microenvironment restrict immune activity. Panel (**B**). Following primary injury or dysfunction of primary afferent fiber activity. Damaged afferents release CCL2 and CX3CL1, activating glia and promoting the release of pro-inflammatory cytokines. Endothelial upregulation of ICAM-1 and PECAM-1 permits T-cell infiltration, while barrier disruption enhances immune cell entry. Neuroinflammation drives central sensitization and neuropathic pain.

The immune system plays a pivotal role in both the initiation and persistence of neuropathic pain, particularly following peripheral nerve injury. Under physiological conditions, the spinal cord exhibits relative immune privilege, maintained by the blood–spinal cord barrier, the scarcity of classical antigen-presenting cells, and an immunosuppressive microenvironment in which astrocytes regulate T-cell activity and dampen inflammatory signaling (Figure 3). Chronic or intense noxious stimuli disrupt this homeostatic equilibrium by increasing blood–spinal cord barrier permeability via downregulation of tight junction proteins and upregulation of matrix metalloproteinases. This facilitates the infiltration of macrophages, peripheral immune cells, growth factors, and soluble mediators into the DH. Concurrently, damaged primary afferent fibers release chemotactic signals such as CCL2 and CX3CL1, which activate microglia and astrocytes, leading to the secretion of pro-inflammatory cytokines and chemokines (IL-1 beta, IL-6, TNF, CCL2) [20]. In parallel, spinal endothelial cells upregulate adhesion molecules (ICAM-1, PECAM-1), promoting transendothelial migration of ITGAL-expressing T cells into the DH. Chemokine gradients and CCL2-driven disruption of tight junctions further amplify barrier breakdown and immune cell entry. The resulting pro-inflammatory environment amplifies central sensitization, sustains immune cell recruitment, and establishes a self-perpetuating cycle of neuroinflammation that contributes to the initiation and maintenance of neuropathic pain.

#### 2.2.3. Neuroendocrine Modulation in the Spinal Cord

Accumulating evidence indicates that the DH is an autonomous neuroendocrine site capable of synthesizing and locally releasing neurosteroids and hormones. These in situ mechanisms regulate nociceptive transmission and drive maladaptive neuronal plasticity underlying neuropathic pain states. Both neurons and glial cells in the spinal cord express key steroidogenic enzymes, enabling local production of pregnenolone, progesterone, and their active metabolites. Additionally, aromatase expression in superficial DH neurons supports local estrogen biosynthesis, further contributing to the neuroendocrine modulation of spinal nociceptive circuits [21]. These neurosteroids modulate specific effects depending on context: estrogens, through membrane-bound estrogen receptors, enhance N-methyl-D-aspartate (NMDA) receptor activity and drive synaptic plasticity linked to pain hypersensitivity [22]. Progesterone and its metabolite allopregnanolone activate antinociceptive pathways by dampening pro-inflammatory cytokine release, suppressing glial activation, and potentiating GABA_A_ receptor signaling [23]. Neuropathic pain leads to marked upregulation of neurosteroidogenesis, suggesting that local steroid production represents an intrinsic adaptive mechanism that counterbalances excitability and inflammation. However, its role may be both protective and maladaptive depending on the balance of estrogenic versus progestogenic actions [24,25].

### 2.3. Synaptic Mechanisms of Nociceptive Transmission

#### 2.3.1. Normal Physiological State

Under physiological conditions, nociceptive processing in the DH depends on a tightly regulated excitatory-inhibitory (E-I) balance that ensures selective encoding of noxious stimuli while preventing aberrant activation by innocuous sensory inputs. Within the DH, excitatory projection neurons and local interneurons form intricate networks that gate nociceptive signals ascending to supraspinal centers. GABAergic and glycinergic interneurons maintain inhibitory tone by modulating synaptic transmission at both presynaptic primary afferent terminals and postsynaptic second-order neurons. Glial cells further contribute to antinociceptive regulation by controlling neuronal excitability and shaping the local inflammatory microenvironment.

**Figure 4:**
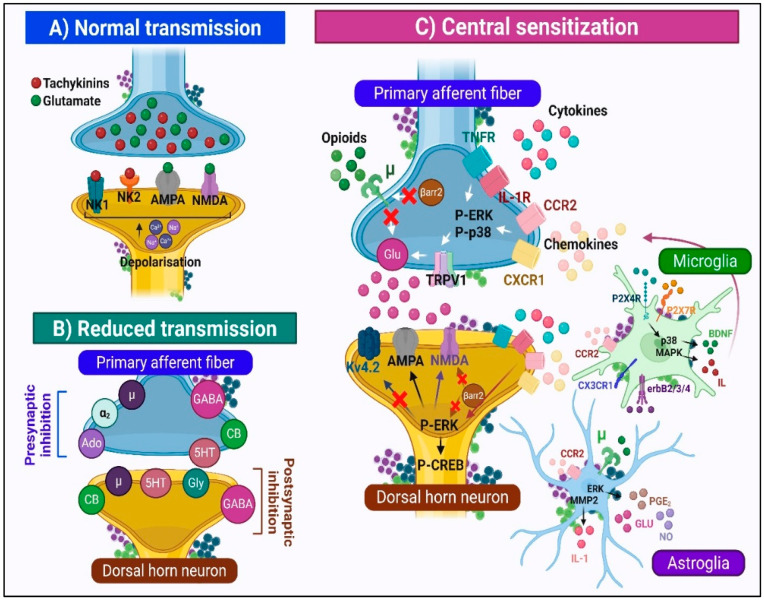
Mechanisms of nociceptive signal transmission and modulation. Panel (**A**). Normal Transmission: Nociceptive impulses trigger vesicular release of glutamate and tachykinins from primary afferent terminals. Glutamate activates AMPA and NMDA receptors. Tachykinins bind NK1/NK2 receptors, activating G-protein signaling. Panel (**B**). Reduced Transmission: Inhibitory modulation operates at presynaptic and postsynaptic levels. Presynaptically, μ-opioid and 5-HT receptor activation reduces glutamate and substance P release. Postsynaptically, GABA_A and glycine receptors open Cl^−^ channels. Panel (**C**). Central Sensitization: Presynaptically, afferents upregulate TRPV1 channels and activate cytokine/chemokine receptors, increasing P-ERK and P-p38 activity and enhancing glutamate release. Neurons release fractalkine (CX3CL1) and CCL2, activating microglial CX3CR1 and CCR2 receptors. Postsynaptically, NMDA Mg^2+^ blockade is relieved, AMPA receptors undergo post-translational modifications, Kv4.2 channels are inhibited, and P-CREB activation drives pro-nociceptive gene transcription. Microglia and astrocytes release pro-inflammatory mediators.

Upon the arrival of nociceptive impulses at the DH, the activation of primary afferent terminals evokes vesicular release of excitatory neurotransmitters, primarily glutamate, as well as neuropeptides such as substance P (SP), neurokinin A, and calcitonin gene–related peptide (CGRP). Glutamate mediates fast excitatory synaptic transmission through ionotropic alpha-amino-3-hydroxy-5-methyl-4-isoxazolepropionic acid (AMPA) and NMDA receptors, both essential for acute and chronic pain modulation. NMDA receptor activation triggers intracellular cascades via calcium influx and the activation of protein kinases, thereby enhancing membrane excitability, synaptic plasticity, and nociceptive transmission. Concurrently, tachykinins such as SP activate neurokinin-1 (NK1) and neurokinin-2 (NK2) receptors, initiating G protein-coupled signaling pathways that sustain postsynaptic depolarization. Intense noxious stimulation induces SP release from dorsal root ganglion terminals, sensitizing DH neurons [5,26,27] (Figure 4).

Under basal conditions, NMDA receptor channels are blocked by extracellular Mg^2+^ ions. Strong or repetitive nociceptive input depolarizes the membrane, relieving this block and enabling Na^+^ and Ca^2+^ influx. Under persistent inflammatory conditions, sustained NMDA receptor activation drives Ca^2+^ entry and PKC-alpha-mediated phosphorylation of GluR-2 at Ser880, disrupting its anchoring and promoting receptor internalization. Elevated intracellular Ca^2+^ subsequently activates PKC and Ca^2+^/calmodulin-dependent protein kinase II (CaMK-II), which further potentiates excitatory neurotransmission through phosphorylation-dependent mechanisms [26].

Multiple endogenous inhibitory neurotransmitters, including gamma-aminobutyric acid (GABA), glycine, norepinephrine, serotonin (5-HT), adenosine, cannabinoids, and opioid peptides, act through their specific receptors to modulate pain [5]. Presynaptically, activation of mu-opioid and 5-HT receptors reduces the release of excitatory neurotransmitters (glutamate and SP) from nociceptive afferents. Endogenous opioids (enkephalins, endorphins, dynorphins) modulate pain via mu-, delta-, and kappa-opioid receptors. Similarly, endocannabinoids (eCBs) provide additional inhibitory regulation by activating cannabinoid receptors. Postsynaptically, the opening of Cl^−^ channels via GABA_A_ and glycine receptors hyperpolarizes the membrane, dampening postsynaptic depolarization (Figure 4). In addition, descending brainstem pathways from the periaqueductal gray and rostral ventromedial medulla modulate spinal nociceptive transmission through noradrenergic and serotonergic signaling, using both inhibitory and facilitatory influences [28].

#### 2.3.2. Central Sensitization: The Transition to Pathological Pain Processing

Central sensitization reflects a state of neuronal hyperexcitability in which central nociceptive pathways present amplified responses to regular or previously subthreshold afferent stimulation. This phenomenon emerges from the convergence of multiple pathological processes: (1) increased membrane excitability of DH nociceptive neurons, facilitating their activation by stimuli that would generally be subthreshold; (2) facilitation of synaptic transmission due to changes in neurotransmitter release and receptor function, as well as synaptic plasticity that enhances neuronal responses; and (3) reduction in inhibitory influences, primarily mediated by GABA and glycine, which diminishes normal inhibitory tone and allows non-nociceptive afferents (e.g., A-betaAβ fibers) to activate nociceptive circuits, leading to allodynia [4,29].

Within this sensitized state, multiple convergent mechanisms act synergistically to amplify nociceptive signaling and sustain neuronal hyperexcitability (Figure 4). Presynaptic inhibition is weakened by the downregulation or desensitization of adenosine, serotonin, mu-opioid, and GABA_A/B_ receptors, thereby diminishing inhibitory modulation of transmitter release [30,31]. Simultaneously, excitatory neurotransmission is amplified by the upregulation of the transient receptor cation channel subfamily V member 1 (TRPV1) channel and the activation of cytokine/chemokine receptors (CCR2, CXCR1, TNFR). These receptors engage extracellular signal-regulated kinase (ERK) and p38 mitogen-activated protein kinase (MAPK) phosphorylation cascades that potentiate glutamate release by increasing the activity of TRPV1, Nav1.7, and Nav1.8 channels [32]. Further presynaptic facilitation occurs via autoreceptor-driven positive feedback at NMDA, P2X, and metabotropic glutamate (mGluR) receptors, reinforcing excitatory transmitter release [33,34].

Increased expression and activity of voltage-gated calcium channels (VGCCs) further augment Ca^2+^ influx, promoting synaptic vesicle fusion and exocytosis [35]. Postsynaptically, activation of AMPA, NMDA, mGluR, and NK1 receptors elevates intracellular Ca^2+^ concentration and activates kinases such as PKC, ERK, and p38 MAPK. These cascades relieve the Mg^2+^ block of NMDA receptors, potentiating excitatory postsynaptic currents. Concomitant modulation of Kv4.2 potassium channels and CREB phosphorylation promotes pronociceptive gene transcription and synaptic remodeling, consolidating long-term potentiation and persistent hyperexcitability [36].

Glial cells, particularly astrocytes and microglia, play a crucial role in the initiation and maintenance of central sensitization [37] (Figure 2). In response to repetitive or intense noxious stimuli, both cell types acquire reactive phenotypes that disrupt neuronal homeostasis. Microglial activation is characterized by morphological changes (microgliosis), upregulation of activation markers (CD11b, P2X4 receptors, TLR4, major histocompatibility complex class II), and phosphorylation of p38 MAPK. This microglial response typically precedes astrocytic activation by several days, suggesting that microglia initiate the sensitization cascade while astroglia sustain chronic hyperexcitability. Activated microglia release pro-inflammatory cytokines and chemokines, while reactive astrocytes upregulate glial fibrillary acidic protein (GFAP) and secrete gliotransmitters. Together, these glial responses intensify nociceptive signaling and lower the pain threshold [15,16]. These responses foster a localized neuroinflammatory environment characterized by leukocyte infiltration, increased vascular permeability, and persistent mediator production [4].

## 3. Spinal Endocannabinoid System: Implications in Neuropathic Pain

**Figure 5:**
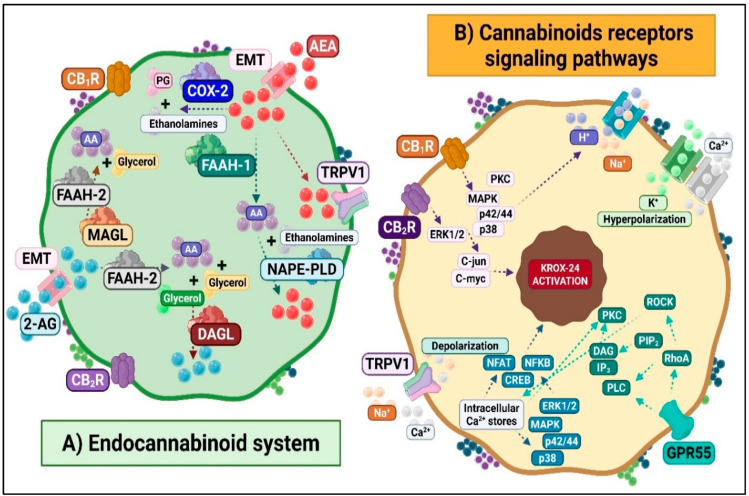
ECS components and cannabinoid receptor signaling pathways. Panel (**A**). Endocannabinoid System. The main endocannabinoids (eCBs) are anandamide (AEA) and 2-arachidonoylglycerol (2-AG). AEA synthesis involves N-acyltransferase (NAT) and NAPE-phospholipase D (NAPE-PLD). 2-AG is synthesized via phospholipase C (PLC) and diacylglycerol lipase (DAGL). FAAH degrades AEA, and MAGL degrades 2-AG; both yield arachidonic acid. Panel (**B**). Cannabinoid receptor signaling. *CB1R* couples to Gi/o proteins, inhibiting adenylyl cyclase, blocking Ca^2+^ channels, opening K^+^ channels and activating MAPK/ERK pathways. *CB2R* shows similar adenylyl cyclase inhibition and MAPK activation without ionic channel modulation. *GPR119* couples to Gs proteins, stimulating cAMP/PKA signaling. *GPR55* couples to G_13_ proteins, activating PLC/RhoA pathways and increasing Ca^2+^ and MAPK/ERK signaling. *TRPV1* activation causes cation influx and depolarization. CB1R/CB2R activate KROX-24 via MAPK/ERK, regulating synaptic plasticity and gene expression.

The endocannabinoid system (ECS) is a versatile lipid-signaling network with a pivotal role in modulating pain processing and neural regulation [38,39]. (Figure 5). Classically, it comprises two G protein-coupled receptors, cannabinoid type 1 (CB1R) and type 2 (CB2R), along with endogenous ligands (eCBs), a membrane transport system, and metabolic enzymes that form and degrade the ligands. Several endogenous eCBs have been identified: anandamide (AEA) and 2-arachidonoylglycerol (2-AG) are the best characterized [40], while others include noladin ether, virodhamine, and N-arachidonoyl dopamine. Structurally related N-acylethanolamines (NAEs), such as N-oleoylethanolamine and N-palmitoylethanolamine (PEA), further expand the biochemical diversity of this system. AEA and 2-AG are made on demand from membrane lipids and rapidly released, acting mainly on CB1R and CB2R. However, they also modulate other molecular targets, including the orphan receptors GPR55 and GPR119, the TRPV1 channel, and nuclear peroxisome proliferator-activated receptors (PPARs) [41,42]. 

Termination of eCB signaling occurs through transport and enzymatic degradation. Fatty acid amide hydrolase (FAAH) predominantly hydrolyzes AEA, and monoacylglycerol lipase (MAGL) mainly degrades 2-AG, yielding arachidonic acid as a standard product.

### 3.1. Molecular and Cellular Responses of the ECS to Noxious Stimuli

At the intracellular level, CB1R activation couples to G_i/o_ proteins, leading to inhibition of adenylyl cyclase, blockade of voltage-sensitive Ca^2+^ channels, enhancement of K^+^ conductance, and stimulation of MAPK/ERK cascades (Figure 5). Together, these mechanisms induce hyperpolarization and regulate neurotransmitter release. CB2R activation similarly inhibits adenylyl cyclase and triggers MAPK activation but lacks direct ionic channel coupling [43]. Other ECS-related receptors trigger distinct signaling pathways. GPR119 couples to G_s_ proteins and enhances cAMP/PKA signaling [44].

In contrast, GPR55 engages G_13_ proteins to activate phospholipase C and RhoA [45]. This activation increases intracellular Ca^2+^ levels and ERK phosphorylation. TRPV1 activation allows non-selective cation influx and depolarization, contributing to nociceptive transmission, while PPAR activation modulates the transcription of genes involved in lipid metabolism and inflammation [46]. Notably, activation of CB1R and CB2R in turn activates the KROX 24 transcription factor via MAPK/ERK cascades, thereby regulating long-term potentiation, cell differentiation, and neurotransmitter/receptor expression (Figure 5).

At the spinal level, ECS components exert dual effects on nociceptive processing, which may be inhibitory or facilitatory [39,47]. Under basal conditions, physiological eCB tone modulates synaptic transmission. However, peripheral injury or inflammation rapidly enhances eCB signaling within hours of onset [48]. This adaptive response modulates multiple processes. These include excitatory and inhibitory neurotransmission, glial responses, and immune interactions [10]. Collectively, these mechanisms shape spinal sensitization and pain outcomes. The complex interplay among CB1R- and CB2R-mediated effects, TRPV1 activation, and PPAR-driven transcription underscores the ECS as a multifaceted regulatory system within the spinal cord, capable of shifting from a protective to a maladaptive mode depending on the timing, location, and nature of neuronal injury.

#### 3.1.1. Spinal ECS and Antinociceptive Mechanisms

The ECS shows extensive antinociceptive efficacy in preclinical models of neuropathic pain [49]. Evidence demonstrates that strategies, including both pharmacological and genetic inhibition of endocannabinoid-degrading enzymes, effectively boost endocannabinoid tone to reduce pain and inflammation. A paradigmatic example is the inhibition or deletion of FAAH, which elevates in vivo levels of AEA and produces pronounced antinociceptive effects in models of inflammatory and neuropathic pain. Furthermore, FAAH inhibition concurrently increases PEA levels. This bioactive lipid mediator exhibits anti-inflammatory and analgesic properties by activating the nuclear receptor PPAR-alpha.

Complementing enzyme-inhibition strategies, synthetic CB1R and CB2R cannabinoid agonists have also demonstrated good efficacy. Intravenous administration of WIN 55,212-2, a non-selective cannabinoid receptor agonist, produces a dose-dependent reduction in the activity of wide-dynamic-range neurons in the DH of rats exposed to chronic constriction injury. Similarly, selective CB2R agonists, such as JWH-133, exhibit antinociceptive effects by attenuating mechanical nociceptive responses through spinal CB2R. Additional therapeutic strategies targeting the endocannabinoid metabolism have yielded promising results. The endocannabinoid uptake inhibitor UCM707 reduces mechanical allodynia in neuropathic pain models through mechanisms involving both CB2R and TRPV1 receptors [50].

At the spinal level, ECS-mediated antinociception operates through three functionally integrated processes: (1) inhibition of ascending nociceptive transmission, (2) enhancement of descending inhibitory modulation, and (3) neuroimmune modulation.

##### Inhibition of Ascending Nociceptive Transmission

**Figure 6:**
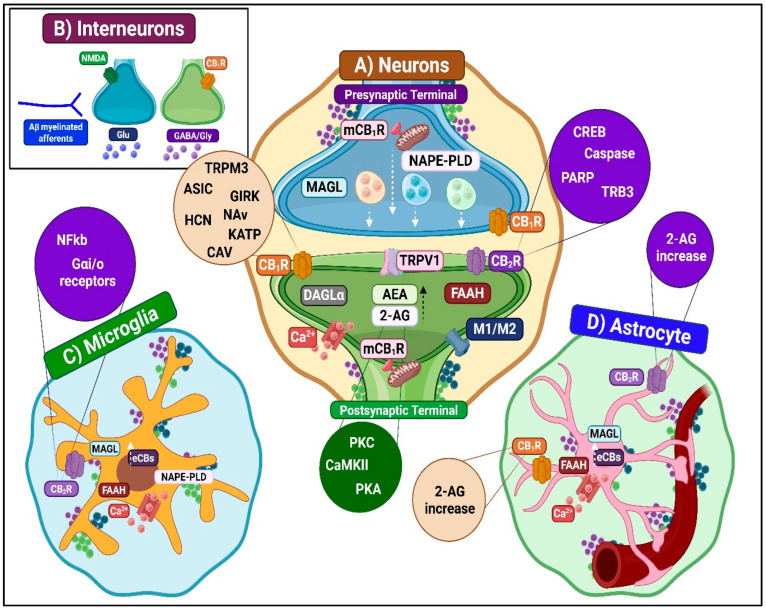
ECS-mediated antinociceptive modulation in the spinal DH. (**A**) Nociceptive projection neurons: Postsynaptic activity induces eCB synthesis. These mediators diffuse retrogradely to presynaptic CB1Rs (Gi/o-coupled), triggering: (i) Gαi/o-mediated adenylyl cyclase inhibition, reducing cAMP/PKA activity and modulating ion channels (TRPV1, HCN, ASIC, Nav); (ii) suppression of VGCCs and TRPM3, decreasing Ca^2+^ influx and neurotransmitter release; (iii) Gβγ-mediated activation of GIRK and K_ATP channels, producing hyperpolarization; (iv) MAPK cascade activation (ERK, JNK, p38) for long-term transcriptional changes. CB2R inhibits adenylyl cyclase, suppresses PKA-NF-κB/CREB signaling, and blocks inflammatory gene expression. (**B**) Interneurons: CB1R reduces glutamate release from excitatory interneurons. CB2R activates Gi/o-PLC-IP3 pathway, elevating Ca^2+^ to promote GABA/glycine release and hyperpolarization. (**C**) Microglia: M2 phenotype releases anti-inflammatory mediators (IL-10, TGF-β) and trophic factors (IGF-1, BDNF), and expresses inhibitory receptors (TREM2, CD200R, CX3CR1) that suppress NF-κB. CB2R activation promotes anti-inflammatory cytokines (IL-4, IL-10, NGF), reduces IL-1β, suppresses ATP/P2X4-BDNF signaling, and downregulates CCR2/CX3CR1. PPAR-γ inhibits M1 polarization and suppresses AP-1, STAT, and NF-κB pathways, thereby reducing iNOS and MMP-9. (**D**) Astrocytes: A2 phenotype forms protective barriers and secretes neurotrophic factors (BDNF, NGF, TGF-β, IL-10). CB1R/CB2R activation amplifies 2-AG/AEA release, suppresses pro-inflammatory cytokines, and reduces oxidative stress. eCB-activated PPARs, GPR55, GPR18, and TRPV1 provide additional anti-inflammatory effects.

ECS activation modulates ascending pain transmission through coordinated molecular mechanisms. These involve CB1R, CB2R, TRPV1, and other receptors (Figure 6). The system produces both acute and long-term antinociceptive effects. Key mechanisms include inhibition of excitatory neurotransmitter release, modulation of ion channel permeability, and regulation of gene expression. These processes occur across nociceptive neurons, inhibitory interneurons, and glial cells [38,51].

- CB1R-mediated inhibition

CB1R is abundantly expressed in the superficial DH and modulates synaptic transmission from both excitatory and inhibitory neurons [52]. Presynaptic CB1R activation inhibits glutamate and neuropeptide release, reducing excitatory postsynaptic potentials and attenuating nociceptive input to higher centers [53]. Postsynaptically, CB1R distribution in lamina II and lamina X supports modulation of local and projection neuron activity. CB1Rs form heterodimers with mu-opioid receptors to potentiate antinociception, prevent opioid tolerance, and modulate TRPV1 function [54]. In interneurons, CB1R effects are context-dependent, reducing GABA/glycine release from inhibitory interneurons or suppressing glutamate release from excitatory interneurons, thereby fine-tuning nociceptive circuit activity and central sensitization [55].

- CB2R-mediated inhibition

CB2R, primarily expressed in activated microglia and to a lesser extent in neurons, is upregulated during neuroinflammation [56]. CB2R activation inhibits adenylyl cyclase via G(i/o) alpha coupling, reducing PKA activity and suppressing pro-inflammatory transcription factors. In neurons, CB2R engagement hyperpolarizes membranes and decreases excitatory neurotransmitter release, directly limiting nociceptive drive [57,58]. In microglia, CB2R stimulation suppresses pro-inflammatory mediator release, attenuates neuroimmune interactions, and dampens central sensitization. Indirect modulation of interneurons via microglial signaling enhances inhibitory neurotransmission in the DH [59].

- TRPV1-mediated inhibition

TRPV1, abundantly expressed in laminae I-II, mediates both pronociceptive and antinociceptive effects depending on context [60]. eCBs such as AEA act on TRPV1 to induce partial calcium influx, trigger the release of beta-endorphin and somatostatin, and promote receptor desensitization, all of which contribute to reduced neuronal excitability [61]. TRPV1-mu-opioid receptor cross-talk preserves opioid sensitivity and enhances analgesia [62]. In interneurons, TRPV1 activation can drive inhibitory neurotransmitter release, reduce excitatory glutamate output, and induce long-term depression at excitatory synapses, supporting homeostatic control of spinal nociceptive circuits.

- Other receptor contributions

PPAR-alpha and -gamma reduce glutamate and SP release, inhibit pro-inflammatory transcription factors, and synergize with cannabinoid and opioid systems to suppress central sensitization [63]. GPR119 modulates nociceptive circuits through multiple G protein signaling pathways and typically exerts an inhibitory effect [64].

##### Enhancement of Descending Inhibitory Modulation

The ECS potently modulates spinal nociceptive processing by enhancing inhibitory circuits within the DH. This involves the direct modulation of GABAergic and glycinergic interneurons and is coordinated with activity in supraspinal structures [65]. The underlying mechanisms are complex. First, while CB1R activation presynaptically inhibits GABA release in various circuits, its effect in pathological pain states is to restore homeostasis by paradoxically mitigating the pathological disinhibition associated with central sensitization. Second, CB2R, scarce in descending pathways under physiological conditions, undergoes significant upregulation in microglia and neurons of these pathways during chronic pain states, inflammation, and following nerve injury, highlighting its role as a disease-modifying target.

Furthermore, the ECS enhances descending inhibitory control by interacting with multiple modulatory pathways, including opioid, noradrenergic, and serotonergic systems [66]. This cross-system integration synergistically amplifies their antinociceptive action. As a result, activating cannabinoid receptors not only provides direct antinociceptive effects but also enhances the efficacy of various endogenous pain-suppressing mechanisms.

##### Neuroimmune Modulation

Glial cells are well-established as central regulators of neuroinflammatory processes, predominantly recognized for their role in amplifying nociceptive signaling in neuropathic pain states [67,68] (Figure 2). However, research reveals their dual function, demonstrating that they also possess antinociceptive capabilities. They promote the resolution of neuroinflammation and the restoration of homeostatic balance within spinal neural circuits [15,16,69]. Within this complex framework, the ECS emerges as a key participant in maintaining immune homeostasis [16,70]. Thus, ECS activation inhibits pro-inflammatory cytokine production and modulates glial cell activity. This exerts immunosuppressive or immunoregulatory effects depending on the physiological context.

- Microglia-dependent antinociceptive mechanisms

Microglia employ dual regulatory influence on pain processing. They adopt either a pro-inflammatory M1 phenotype that amplifies nociception or an anti-inflammatory M2 phenotype that promotes analgesia and tissue repair [15,69]. In their M2 state, microglia attenuate pain through multiple mechanisms. They release anti-inflammatory cytokines such as IL-10 and transforming growth factor (TGF)-beta [71,72]. Additionally, enhanced phagocytic clearance of cellular debris contributes to the resolution of neuroinflammation [73]. The expression of inhibitory receptors, including TREM2, CD200R, and CX3CR1, further suppresses NF-kappa-B–dependent transcription of pronociceptive genes [74]. This microglial modulation extends to synaptic plasticity via the secretion of trophic factors (insulin-like growth factor 1, brain-derived neurotrophic factor [BDNF]) [75,76], which is complemented by adenosine-mediated purinergic signaling through A2A and A3 receptors [77]. These receptors reinforce the anti-inflammatory phenotype and provide direct analgesic effects.

Microglia represent the principal source of endogenous cannabinoid synthesis (Figure 6). CB2R activation stimulates the secretion of IL-4, IL-10, and nerve growth factor (NGF) while simultaneously suppressing IL-1 beta release and ATP/P2X4 signaling [78]. These effects limit neuroinflammation and microglial overactivation.

The presence of functional CB1R in microglia implies a potential antinociceptive role for these glial receptors [79]. Alternatively, observed atypical cannabinoid responses may be mediated by orphan receptors, such as GPR55, which are expressed in microglia and can explain specific CB1R/CB2R-independent effects [80].

- Astrocyte-dependent antinociceptive mechanisms

Under physiological conditions, astrocytes are fundamental for maintaining synaptic homeostasis by providing structural and metabolic support to neurons. Following nerve injury or persistent noxious stimulation, these cells transition into a reactive state known as astrogliosis. This process involves long-lasting alterations in morphology, function, and gene expression [16]. Unlike microglia, astrocytic activation occurs gradually, beginning 3–7 days post-injury and persisting for weeks to months. This prolonged activation underscores their role in maintaining chronic pain states [68].

Reactive astrocytes present functional heterogeneity, often classified into distinct phenotypes: A1 astrocytes display neurotoxic properties that actively promote chronic pain progression, while A2 astrocytes demonstrate neuroprotective functions by secreting neurotrophic factors that facilitate neuronal survival and tissue repair [68]. This highlights the dual nature of the astroglial response. In their protective capacity, reactive astrocytes contribute to glial barrier formation, restricting the spread of inflammatory damage. They secrete vital neuroprotective factors, including BDNF and NGF, TGF-beta, neurotrophins, and anti-inflammatory cytokines such as IL-10, which collectively facilitate homeostatic balance and limit tissue damage [16]. These actions position astrocytes as central regulators of neuroprotection in the injured nervous system [81].

ECS activation in spinal astrocytes contributes to antinociception by regulating neuroinflammation and modulating synaptic activity [82] (Figure 6). Furthermore, activation of CB1R and CB2R suppresses the release of pro-inflammatory cytokines and promotes immune homeostasis [83]. In addition, astrocytes express peroxisome proliferator-activated receptors (PPAR-alpha, -beta, -gamma), which, upon activation by eCBs, regulate gene expression to reduce oxidative stress and inflammation. Other receptors, such as GPR55, GPR18, and TRPV1, also contribute to anti-inflammatory signaling pathways.

#### 3.1.2. Spinal ECS and Pronociceptive Mechanisms

The ECS is predominantly recognized for its antinociceptive properties; however, it can paradoxically promote nociception under certain conditions [84]. Accordingly, preclinical models of neuropathic pain demonstrate that eCB signaling via CB1R and CB2R can produce opposing effects depending on context and circuit. Moreover, following peripheral nerve injury, spinal cord eCB concentrations increase, particularly AEA [85], leading to activation of CB1R and pronociceptive targets such as TRPV1 channels [86]. This functional shift facilitates the activation of pain signaling pathways.

At the spinal level, ECS-mediated pronociception operates through three functionally integrated processes: (1) potentiation of ascending nociceptive transmission, (2) enhancement of descending excitatory modulation, and (3) neuroimmune modulation.

##### Potentiation of Ascending Nociceptive Transmission

- CB1R-mediated pronociception

**Figure 7:**
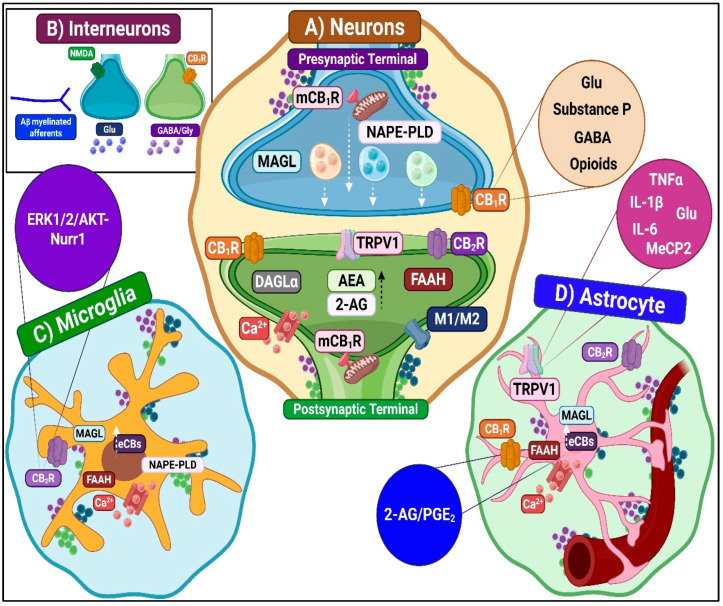
ECS-mediated pronociceptive modulation in the spinal DH. (**A**) Nociceptive projection neurons: CB1R activation facilitates substance P release from primary afferents, evidenced by NK1 receptor internalization. Prolonged CB1R stimulation induces receptor desensitization and downregulation, diminishing antinociceptive capacity. TRPV1 activation in nociceptive neurons triggers Ca^2^-dependent signaling pathways (CaMKII, PKC, MAPK/ERK), enhancing channel phosphorylation and promoting NF-κB-dependent production of pro-inflammatory mediators. (**B**) Interneurons: Activation of CB1R on inhibitory interneurons reduces GABA/glycine release, thereby inducing synaptic disinhibition. TRPV1 activation on GABAergic interneurons triggers Ca^2^-dependent LTD of excitatory inputs, reducing inhibitory tone onto STT projection neurons and facilitating nociceptive transmission. (**C**) Microglia: M1 phenotype release pronociceptive mediators (TNF-α, IL-1β, IL-6, BDNF, CCL2, CXCL1) that enhance dorsal horn neuron excitability. P2X4/P2X7 and TLR4 overexpression, along with intracellular cascades (p38 MAPK, ERK1/2, JNK, NLRP3), consolidate the M1 phenotype. Pathological CB2R activation induces desensitization and impaired phagocytosis via ERK/AKT-Nurr1. Microglial TRPV1 activation promotes ROS production and upregulation of IL-1β and TNF-α. (**D**) Astrocytes: A1 phenotype upregulate inflammatory mediator receptors, enhance connexin-43 gap junctions, downregulate GLT-1, causing glutamate accumulation, and release pronociceptive mediators. Astrocytic CB1R and TRPV1 activation elevate intracellular Ca^2+^, triggering the release of gliotransmitters and pro-inflammatory mediators.

CB1R facilitates nociceptive transmission through disinhibitory mechanisms by enhancing SP release from primary afferent terminals. Activation of CB1R on GABAergic and opioidergic interneuron terminals inhibits GABA and endogenous opioid release, reducing the suppression of SP. The result is increased internalization of NK1 receptors on lamina I projection neurons, suggesting enhanced pronociceptive signaling. CB1R antagonists block, while agonists accentuate this effect, confirming the disinhibitory mechanism [87]. Additionally, prolonged CB1R stimulation induces receptor desensitization and downregulation, diminishing the system’s antinociceptive capacity over time [88] (Figure 7). Moreover, CB1R activation on inhibitory interneurons reduces GABA and glycine release, weakening inhibitory control over second-order nociceptive neurons and promoting hyperexcitability underlying central sensitization. Critical disinhibition of PKC-gamma+ excitatory interneurons in inner lamina II creates a functional “bridge” that enables tactile A-beta fiber input to enter nociceptive pathways, promoting allodynia [3].

- CB2R-mediated pronociception

CB2R expression increases in projection neurons and interneurons during neuropathic pain. However, claims of CB2R-mediated pronociception remain highly debated [59]. The overwhelming scientific consensus supports CB2R as an antinociceptive and anti-inflammatory modulator, primarily acting on glial cells to reduce pain sensitization [80].

- TRPV1-mediated pronociception

TRPV1 channels are established drivers of central sensitization [89]. Their upregulation within the spinal cord enhances receptor sensitivity to normally innocuous stimuli. Crucially, this upregulation also promotes interactions with eCBs, facilitating pronociceptive signaling. Activation of TRPV1 channels in spinal nociceptive neurons induces calcium-dependent signaling cascades involving CaMK-II, PKC and MAPK/ERK pathways [90]. These events strengthen NMDA receptor activation and promote long-term potentiation-like plasticity in DH circuits [33]. TRPV1 activation also induces NF-kappa-B–mediated expression of pro-inflammatory mediators, reinforcing central sensitization [91]. Presynaptically, inflammatory mediators such as TNF enhance glutamate release via TRPV1 sensitization, contributing to mechanical allodynia through aberrant TRPV1 expression in uninjured A-beta fibers. Conversely, TRPV1-positive C and A-delta fibers primarily mediate mechanical hyperalgesia. Postsynaptic TRPV1 activity further sustains central sensitization. It promotes excitatory signaling and disinhibition by stimulating eCB synthesis and activating CB1R. Moreover, TRPV1 channels expressed in spinal GABAergic interneurons induce long-term depression of excitatory synapses. They also reduce inhibitory control over spinothalamic projection neurons, thereby exacerbating central sensitization [92].

- Contributions from other receptors

GPR55 primarily acts as a pronociceptive receptor in the spinal cord, promoting the transmission and maintenance of neuropathic and inflammatory pain. Experimental studies demonstrate that spinal GPR55 activation contributes to mechanical hypersensitivity, neuroinflammation and immune cell recruitment following nerve injury. Conversely, its inhibition or genetic deletion reduces allodynia and inflammatory responses [64].

##### Enhancement of Descending Excitatory Modulation

The ECS paradoxically acts as a pain facilitator through multiple central mechanisms. Under neuropathic pain conditions, the ECS amplifies descending excitatory modulatory pathways. This mechanism occurs by activating CB1R and facilitating “ON-cells” in the rostral ventromedial medulla, increasing their firing frequency. The result is enhanced descending excitatory drive onto spinal nociceptive neurons [93].

Enhanced central activity of the TRPV1 channel is sustained by a descending serotonergic pathway arising from the brainstem and involving 5-HT3A receptors in the DH. This interaction contributes to a facilitation loop that promotes persistent nociceptive transmission. Experimental studies in animal models demonstrate that central pharmacological blockade of TRPV1 or 5-HT3A receptors markedly reduces pain hypersensitivity, supporting the potential clinical relevance of this mechanism [94].

##### Neuroimmune Modulation

- Microglia-dependent pronociceptive mechanisms

Following injury, microglia rapidly adopt a pro-inflammatory phenotype characterized by upregulation of major histocompatibility complex (molecules and the release of key cytokines, including TNF, IL-1 beta, and IL-6). They also secrete chemokines (e.g., CCL2, CXCL1) and neuroactive mediators, including BDNF, nitric oxide, and prostaglandins. These substances enhance the excitability of DH neurons while promoting the recruitment and activation of additional glial cells, thereby sustaining chronic neuroinflammation [95]. Concurrently, pathological overexpression of purinergic receptors (P2X4, P2X7) and pattern recognition receptors (TLR4) occurs, along with activation of intracellular signaling cascades, including p38 MAPK, ERK, JNK, and NLRP3 [14,95]. Together, these processes amplify microglial reactivity and perpetuate maladaptive neuroglial feedback loops.

Under these conditions, cannabinoid receptor activity, mainly through CB2R, can engage pronociceptive mechanisms. Although CB2R generally induces anti-inflammatory effects, sustained or aberrant activation may lead to adverse outcomes. This can lead to receptor desensitization, impaired microglial phagocytosis, and heightened reactivity via ERK/AKT-Nurr1 signaling [96]. Furthermore, paradoxical effects have been observed in specific contexts. CB2R activation can facilitate microglial activation and morphine tolerance in pain and cancer models [97].

Additionally, CB2R signaling may exacerbate neuroinflammation by attracting immune cells across a compromised blood–brain barrier, while TRPV1 activation initiates calcium-dependent intracellular signaling cascades that promote the production of pro-inflammatory cytokines, including IL-1 beta and TNF. These processes collectively contribute to central sensitization and the pathological amplification of pain signaling.

- Astrocyte-dependent pronociceptive mechanisms

Several key astrocytic mechanisms regulate central sensitization and the persistence of neuropathic pain. Upregulation of receptors for inflammatory mediators such as IL-1 beta, TNF, and CCL2 amplifies pro-inflammatory signaling and increases neuronal excitability by altering neurotransmitter release and reuptake [98]. Alterations in gap junction proteins, particularly connexin-43 (Cx43), strengthen intercellular communication among astrocytes [99]. Downregulation of glutamate transporters, especially GLT-1, reduces glutamate clearance, leading to extracellular accumulation. Activated astrocytes also release a wide array of pronociceptive mediators, including chemokines (e.g., CCL2, CXCL1), cytokines (e.g., IL-1 beta, TNF), prostaglandins, and excitatory amino acids such as glutamate [100]. These molecules act on neurons and other glial cells to promote maladaptive synaptic plasticity, enhancing excitatory transmission and reducing inhibitory signaling. CB1R activation in astrocytes increases intracellular calcium levels, triggering the release of gliotransmitters such as glutamate, ATP, and D-serine [82,101]. These molecules enhance excitatory synaptic transmission and reinforce neuron-glia coupling, contributing to spinal hyperexcitability. Moreover, activation of astrocytic TRPV1 receptors exerts pronociceptive effects through calcium-dependent pathways that promote central sensitization and sustain chronic pain states [102].

### 3.2. Determinants in Endocannabinoid System Plasticity

Pain emerges through complex interactions among metabolic, inflammatory, and neurological processes. These collectively modulate neuronal function through two primary mechanisms. First, enhanced membrane excitability is characterized by spontaneous action potential generation, reduced activation thresholds to peripheral stimuli, and expanded receptive fields. Second, augmented inhibitory mechanisms regulate excitability by maintaining a delicate balance between antinociceptive processes and sensitization, ultimately determining perceived pain intensity.

Persistent nociceptive stimulation is critical for ECS plasticity [38,84]. In the acute phases of injury or inflammation, the ECS attenuates pain perception and suppresses neural hyperexcitability. However, prolonged or repetitive nociceptive stimulation can lead to ECS desensitization or dysregulation, compromising its protective function and potentially promoting the development of chronic pain states [103].

To characterize ECS plasticity determinants and cannabinoid agonist responsiveness variability, three key factors warrant consideration: (1) heterogeneity in endogenous and exogenous ligand concentrations, (2) differential spatiotemporal distribution of activated receptors, and (3) variability in cannabinoid-mediated signaling cascades resulting from dynamic changes in ECS receptor expression and metabolic enzyme activity.

#### 3.2.1. Diversity of the Ligand Concentration

The precise balance of endogenous ligands is a critical determinant of the ECS nociceptive response. Current evidence indicates that the ECS undergoes dynamic changes in the spinal cord and associated structures during inflammatory and neuropathic pain. ECBs such as AEA and 2-AG, as well as related NAEs such as PEA, exhibit pathology-dependent fluctuations in concentration [104,105]. These variations are linked to alterations in the activity of biosynthetic (e.g., N-acyltransferase, phospholipase D) and catabolic enzymes (e.g., FAAH, MAGL), which collectively reshape eCB tone under different pain conditions [106].

In models of neuropathic pain, spinal and dorsal root ganglion levels of AEA and 2-AG consistently increase, supporting the ECS’s role as an endogenous modulator of neuronal hyperexcitability and central sensitization [40,107]. Interestingly, AEA tends to rise earlier after nerve injury. At the same time, 2-AG increases at later stages, suggesting a temporal and receptor-specific regulation: AEA predominantly activates CB1R and TRPV1 channels, while 2-AG engages both CB1R and CB2R once glial CB2R expression is upregulated. In contrast, PEA levels often decline in the spinal cord during neuropathic states, which may contribute to heightened pain sensitivity given its intrinsic anti-inflammatory actions [108].

Glial cells, including both microglia and astrocytes, serve as central regulators of the spinal ECS by actively synthesizing and degrading eCBs, particularly during neuroinflammatory states [70,109]. Microglia, for instance, utilize P2X7 receptor-mediated Ca^2+^ influx to drive sustained 2-AG production via activation of diacylglycerol lipases, while also expressing catabolic enzymes such as FAAH, MAGL, ABHD6, and ABHD12 [110].

Astrocytes also express ECS enzymes, and astrocyte-specific MAGL plays a distinct role from neuronal MAGL by hydrolyzing 2-AG to arachidonic acid, linking ECS activity to prostaglandin synthesis and neuroinflammatory processes [111]. This intricate metabolic balance between eCB production and inactivation determines their tissue concentration and subsequent receptor activation.

#### 3.2.2. Diverse Distributions of Activated Receptors

In neuropathic pain models, expression of cannabinoid receptors is altered in primary sensory afferents and postsynaptic spinal cord cells. Spinal administration of the CB1R antagonist AM251, but not the CB2R antagonist SR144528, significantly facilitates mechanically evoked responses in neuropathic rats. Interestingly, the CB2R and endocannabinoid transport modulator, UCM707, did not alter spinal eCBs levels but potently inhibited these responses exclusively in neuropathic rats. Pharmacological studies confirmed that the selective inhibitory effects of UCM707 were mediated by the activation of spinal CB2R and the involvement of TRPV1 channels [112].

These changes occur through alterations in glial receptor expression, as microglia, astrocytes, and oligodendrocytes all express synthetic machinery and receptors [79]. Furthermore, the ECS interacts with other pronociceptive pathways, particularly the TRPV1 feedback loop, which is amplified by elevated anandamide levels, thereby initiating a feed-forward mechanism that includes cyclooxygenase-2 upregulation and enhanced prostaglandin synthesis [113,114]. This process creates a self-amplifying cycle in which TRPV1 activation induces anandamide synthesis, further activating TRPV1 through intracellular calcium-dependent pathways [115]. Additionally, ECS dysregulation can negatively impact descending pain modulation from structures such as the locus coeruleus and the periaqueductal gray [107,116]. Consequently, changes in the CB1R/CB2R expression ratio in neurons and glia can shift the balance from an antinociceptive to a pronociceptive state, demonstrating that receptor distribution is a critical determinant of the ECS response in the spinal cord.

#### 3.2.3. Diversity of Cannabinoid-Mediated Signaling Secondary to Altered ECS Receptor and Enzyme Expression

The modulatory systems dependent on G protein-coupled receptors (GPCRs) and TRPV1 channels exhibit remarkable complexity [117,118]. Individual cells can produce diverse responses depending on the specific receptor activated and the physiological or pathological context in which they are located. Moreover, neurons and glia display distinct release patterns that rely on combinations of GPCRs, including cannabinoid receptors, and their interactions with TRPV1 channels [60].

##### Signaling Related to GPCRs

GPCRs are remarkably versatile signaling proteins that orchestrate intricate intracellular cascades. Over the past decade, growing evidence has revealed additional, and often independent, mechanisms that significantly expand the complexity of GPCR-associated signaling [119]. This diversity arises from multiple interconnected molecular processes that can profoundly alter receptor coupling to G proteins and other intracellular pathways, enabling specialized, regulated and context-dependent cellular responses.

GPCRs were once thought to activate a single canonical pathway; however, it is now well established that the same ligand or pharmacological agent can simultaneously engage multiple pathways, sometimes producing divergent or even opposing cellular effects. This structural flexibility also allows ligands to interact with both orthosteric (primary binding) and allosteric sites, or to promote coupling to different G proteins, thereby expanding the signaling repertoire of a single receptor [117].

Complementing their conformational flexibility, GPCR dimerization and oligomerization establish an additional regulatory framework that further multiplies signaling diversity. These processes can alter the dynamics of G protein coupling and downstream signaling, leading to more specialized and context-dependent cellular responses [120]. CB1R and CB2R exemplify this phenomenon. They can form homodimers (CB1R–CB1R, CB2R–CB2R) as well as heterodimers with each other or with receptors from different systems, including dopaminergic, opioid, orexin, and serotonergic receptors. Notably, CB1R-delta-opioid receptor heteromers have been shown to alleviate chemotherapy-induced neuropathic pain [120], while CB1R-mu-opioid receptor heteromers produce strong antinociceptive effects by inhibiting pain transmission [121].

Another key source of signaling diversity lies in the availability of multiple G protein subtypes, such as G(s) alpha, G(i/o) alpha, and G(q/11) alpha. For example, CB1R activation can inhibit adenylyl cyclase through G(i/o) alpha subunits while simultaneously activating MAPK family members via G beta and gamma subunits. In addition, beta-arrestins extend GPCR signaling beyond their classical role in desensitization and internalization [122,123]_._

##### Signaling Related to TRPV1 Channels

As a key component of the extended ECS, TRPV1 engages in sophisticated molecular interactions with eCBs to regulate the pathophysiology of neuropathic pain. This relationship is exemplified by AEA, which functions simultaneously as a partial CB1R agonist and a direct activator of the TRPV1 channel. The ECS also regulates the expression and function of TRPV1. Specifically, CB1R activation suppresses TRPV1 activity at both the transcriptional and translational levels by reducing intracellular cAMP and PKA signaling, thereby attenuating nociceptive transmission. Moreover, TRPV1 contributes to endocannabinoid degradation via enzymes such as FAAH, while FAAH inhibitors elevate spinal AEA levels, producing antinociceptive effects through progressive TRPV1 desensitization [124].

## 4. Therapeutic Implications

The complex crosstalk among eCBs, microglial activation, and CB2R signaling exemplifies the complexity of pain processing, which involves context-dependent functional plasticity oscillating between pain-promoting and pain-relieving states. Factors include the local neurochemical microenvironment, microglial polarization phenotypes (M1 vs. M2), and the degree of synaptic neuroplasticity within spinal DH circuits. This dynamic behavior fundamentally contradicts the simplistic view that cannabinoids are universally anti-inflammatory or analgesic. Consequently, the therapeutic efficacy of cannabinoids is tightly linked to specific pain pathophysiology. This recognition underscores the need for precision cannabinoid-based therapeutics, based on the development of biomarker platforms to characterize endogenous ECS function, define glial activation states, and quantify differential cannabinoid receptor expression in nociceptive target tissues.

### 4.1. General Considerations

#### 4.1.1. Use of the Intrathecal Route

The intrathecal route is a therapeutically advantageous strategy for managing neuropathic pain, enabling direct drug delivery into the cerebrospinal fluid [125]. This approach bypasses the blood–brain barrier, facilitating optimized interactions with endogenous modulatory systems within the spinal DH. Key clinical benefits include the interruption of pain transmission at the spinal level, reduction in nociceptive input to supraspinal centers, and the requirement for significantly lower drug doses compared to systemic administration. These attributes collectively contribute to a marked decrease in systemic side effects.

Nevertheless, it is crucial to clarify that within the scope of this research, the intrathecal route is employed as an experimental model rather than a clinical recommendation. While intrathecal administration is a well-established route in pain neuroscience, essential for exploring spinal mechanisms without the confounding influence of supraspinal or systemic processes, and despite intrathecal administration via implantable pumps being an established therapeutic option for patients with neuropathic pain refractory to conservative measures [126,127], this therapeutic modality is integrated in a limited capacity within the classical intervention model. Specifically, the intradural route is not part of routine primary care practice. It is typically considered a "critical mass" strategy, viable only in specialized tertiary care centers that possess the necessary resources and adequately trained personnel. 

Although these practical limitations exist, intrathecal cannabinoid agonists effectively induce antinociception. Robust evidence from behavioral, electrophysiological, and neurochemical studies demonstrates that cannabinoids modulate nociceptive processing directly within the spinal cord. Mixed agonists such as levonantradol, WIN55,212-2, and CP55,940 exhibit significant antinociceptive effects, further supporting the therapeutic potential of spinal cannabinoid delivery [128,129]. Additionally, spinal administration of inhibitors targeting endocannabinoid-degrading enzymes, such as FAAH (e.g., URB597, AA-5-HT) and MAGL (e.g., URB602), enhances endocannabinoid-mediated stress-induced analgesia [124].

#### 4.1.2. Manipulation of Endocannabinoid Levels and Adaptive Responses to Injury

Therapeutic manipulation of the ECS in neuropathic pain, especially at the spinal level, requires a comprehensive understanding of the adaptive and plastic processes the system undergoes following injury (see the Section 3.2). The response to neural damage is intrinsically dynamic, showing significant variability in both magnitude and temporal kinetics. Beyond merely activating cannabinoid receptors, current strategies aim to precisely modulate endogenous endocannabinoid levels through targeted enzyme inhibition, selective engagement of receptor subtypes such as CB2R, and intervention in the formation and function of supramolecular complexes (e.g., heteromers).

A representative example of ECS adaptive complexity is the desensitization and downregulation of the CB1R. This process reduces its sensitivity and functional expression following sustained or excessive stimulation or prolonged treatment. This adaptive regulation is accompanied by critical temporal differences in the activation of intracellular signaling cascades, including the ERK pathway, as well as other molecular mechanisms associated with cannabinoid receptors.

Simultaneously, glial cells display systematic phenotypic and functional heterogeneity depending on the specific type of neural injury, the precise anatomical location, and the temporal stage of the pathology [130]. Traditional dichotomous classification paradigms (e.g., pro-inflammatory M1 vs. reparative M2 for microglia; neurotoxic A1 vs. neuroprotective A2 for astrocytes) are outdated. More sophisticated conceptual models now recognize a continuous spectrum of glial activation states with dynamic and mixed functional properties, reflecting the complexity of their roles in the nervous system [131].

Consequently, therapeutic manipulation of the ECS must consider not only the rational selection of specific molecular targets but also the optimal timing of intervention and the characterization of the prevailing pathophysiological context. Such a multifaceted approach aims to maximize desired analgesic effects, minimizing adverse outcomes and counterproductive adaptations that could compromise therapeutic efficacy.

#### 4.1.3. Therapies Targeting Glial Cells

Central sensitization in neuropathic pain involves crucial contributions from glial cells, particularly microglia and astrocytes [14,100]. Following nerve injury, the activation of these cells initiates a neuroinflammatory cascade that sustains and amplifies central sensitization. Glia not only modulate the synaptic microenvironment but also actively secrete a diverse range of pro-inflammatory mediators. These factors induce maladaptive neuroplastic changes within the central nervous system. This understanding recasts glial cells from mere support cells to active participants, underscoring their pivotal role in the onset, amplification, and perpetuation of neuropathic pain. Therefore, therapeutic strategies targeting glial activation and signaling pathways hold promise for disrupting these critical non-neuronal mechanisms and improving neuropathic pain management.

#### 4.1.4. Multimodal Analgesia

The complex nature of neuropathic pain has led to the development of multimodal therapeutic strategies that combine different analgesic therapies. These interventions are based on the idea that simultaneously targeting multiple pathways can more effectively suppress neuroinflammation and provide better pain relief than single-agent treatments [10,132].

A promising therapeutic approach involves coadministration of p38 MAPK inhibitors, key mediators of microglial activation and pro-inflammatory cytokines IL-1 beta, TNF, and IL-6, with opioid agonists. This combination significantly enhances antinociception, delays tolerance onset, and mitigates adverse effects associated with prolonged opioid use [133].

Another strategy is combining cannabinoids and opioids. CB1R, CB2R, and mu-opioid receptors are all present in the spinal DH. Combining these agents allows for a dose reduction of each while still maintaining, or even enhancing, pain relief and lowering the risk of adverse effects [134,135].

A third approach pairs FAAH and MAGL enzyme inhibitors (e.g., URB597 and JZL184) with glial modulators. This combination works by naturally and sustainably increasing the levels of the eCBs anandamide and 2-arachidonoylglycerol, which boosts the ECS while reducing inflammation in glial cells [124]. The resulting long-lasting pain relief shows significantly less tolerance compared to direct use of synthetic cannabinoid agonists. Additionally, simultaneous inhibition of microglia and astrocytes with agents such as minocycline and propentofylline provides a broader blockade of spinal inflammatory responses, helping restore normal neuronal function [68,100] (See Table 1).

### 4.2. Specific Considerations

#### 4.2.1. ECS-Related Strategies

##### Spinal CB1R-Mediated Modulation

Accumulating research has progressively elucidated the fundamental role of CB1R in analgesic processes and the complex CB1R-mediated mechanisms that regulate neuropathic pain [55]. CB1R receptors in primary sensory neurons of the DRG play a crucial role in controlling the cellular and molecular processes underlying neuropathic pain [136,137]. In this sense, different strategies targeting CB1R have been explored as potential treatments for this condition.

To evaluate the effects of spinal CB1R activation, studies were conducted using the CB1R agonist ACEA, administered intrathecally. First, an experimental model of streptozotocin-induced diabetic neuropathic pain in rats was employed to confirm that activation of the CB1R at the spinal level significantly improves mechanical allodynia. Notably, the anti-allodynic effect of CB1R was reversed by the prior intrathecal administration of AM251, a selective CB1R antagonist. This finding reveals the critical and specific role of CB1R in ACEA-induced analgesia [138]. Second, a rat neuropathic pain model induced by chronic constriction injury of the sciatic nerve was employed to investigate the effect of intrathecal ACEA injection on the expression and function of hyperpolarization-activated cyclic nucleotide-gated (HCN) channels, types 1 and 2 (HCN1/2), in the DRG.

ACEA significantly decreased mechanical allodynia and reduced the expression of HCN1 and HCN2 channels. This effect was blocked by AM251, suggesting that CB1R activation can inhibit the HCN channel’s function [139].

Electrophysiological studies are crucial for elucidating the mechanisms underlying spinal nociceptive transmission. Employing a spinal nerve ligation model in mice and in vitro patch-clamp recording of the C-fiber evoked excitatory postsynaptic currents (C-eEPSCs), in vivo extracellular recording of spinal local field potential, and a mechanical hypersensitivity paradigm (i.e., von Frey test), revealing that CB1R blockade by intrathecal pretreatment with AM251 reduced the inhibition of C-eEPSCs in excitatory neurons and inhibitory GABAergic interneurons located in the substantia gelatinosa of the spinal cord.

Intrathecal administration of tetrahydrocannabinol (THC) has also been explored in different animal models of nociceptive and neuropathic pain. In standard formalin and complete Freund’s adjuvant tests of nociceptive and inflammatory pain, intrathecal THC injection was explored in several genetically modified mice devoid of CB1R, CB2R, and Cav3.2 T-type Ca^2+^ channel isoform [140]. Data revealed that Cav3.2 channels are essential targets for the analgesic actions of delta_9_-THC, whereas CB1R and CB2R are not required under these experimental conditions [141].

Experiments have also been conducted with other molecules showing therapeutic potential for neuropathic pain, identifying CB1R as a key target in their mechanism of action. For instance, kavalactone yangonin, isolated from *Piper methysticum* and commonly known as kava, was administered intrathecally to evaluate its anti-hyperalgesic and anti-allodynic actions in carrageenan-induced paw inflammation and a partial spinal nerve ligation model in rats, respectively. Yangonin did not affect mechanical allodynia at the spinal level; however, it exhibited a relatively potent antinociceptive effect, attenuating carrageenan-induced hyperalgesia. Furthermore, another study investigated the potential antinociceptive effect of aspirin-triggered lipoxin A_4_ (ATL), an endogenously produced anti-inflammatory lipid mediator, when administered alone or in combination with the CB1R agonist ACEA in a streptozotocin-induced diabetic neuropathic pain model in rats [142].

##### Spinal CB2R-Mediated Modulation

CB2R shows significant promise for the treatment of neuropathic pain. Preclinical studies have consistently demonstrated that activation of CB2R produces antinociceptive and anti-hyperalgesic effects in models of neuropathic pain [56]. This therapeutic benefit stems from the fact that CB2R is mainly expressed in immune and glial cells, making it an attractive and safer target for treatment [43,55].

Notable advantages of CB2R agonists include their superior safety profile, characterized by the absence of central nervous system adverse effects typically associated with CB1R-targeting compounds [143]. Furthermore, CB2R activation shows resistance to analgesic tolerance and effectively inhibits key mechanisms of central sensitization that underlie chronic neuropathic pain states [57,144,145,146].

The development of CB2R-selective cannabinoid agonists has yielded diverse chemical classes with significant therapeutic potential for pain management. Compounds derived from delta_9_-THC and -delta_8_-THC, as well as phytocannabinoid analogs such as HU-308, exhibit varying degrees of CB2R selectivity [81]. The tricyclic cannabinoid class includes octahydrophenanthridines such as L-759,632, which exhibits 305-fold CB2R selectivity, along with AM1710 (750-fold selectivity) and AM724 (407-fold selectivity). Notably, the synthetic benzoquinolinone derivative Sch35966 displays exceptional selectivity, and even natural ligands, such as alkylamides from *Echinacea* spp., act as cannabinomimetics with greater affinity for CB2R.

##### Spinal Non-CB1R/CB2R Cannabinoid Receptors Modulation

Spinal TRPV1 channels are compelling therapeutic targets for neuropathic pain treatment, spanning selective antagonists, negative allosteric modulators, inhibition of phosphorylation cascades, promotion of TRPV1 internalization, and reduction in glial neuroinflammation that sensitizes these channels [60]. Of particular interest among the treatments being investigated is the intrathecal administration of TRPV1 siRNA (small interfering RNA), which is specifically designed to silence *TRPV1* gene expression. This method has been shown to effectively reduce peripheral neuropathy and pain sensitivity by suppressing *TRPV1* expression in the spinal cord. This therapeutic approach also reduces mechanical sensitivity caused by paclitaxel-induced neuropathy, highlighting its potential as a targeted treatment for various types of neuropathic pain [147]. Moreover, intrathecal administration of TRPV1 antagonists shows antinociceptive effects [148]. However, for clinical use, TRPV1 modulators face several challenges. Antagonists may lead to side effects related to body temperature regulation.

Additionally, their adverse effects have not been thoroughly evaluated in preclinical studies. A promising solution involves combining TRPV1 modulation with regulators of the endocannabinoid system. This integrated approach can enhance spinal pain relief while minimizing side effects and the risk of central sensitization.

Recent investigations have examined the role of GPR55 in nociceptive modulation, revealing complex, sometimes paradoxical effects. In the brain’s descending pain control centers, specifically the periaqueductal gray and the rostral ventromedial medulla, GPR55 seems to induce a pronociceptive effect. Accordingly, the administration of ML-193, a selective GPR55 antagonist, into these pain control centers produced a significant anti-allodynic impact, supporting the idea that blocking GPR55 in these areas alleviates pain [149].

In contrast, at the spinal level, GPR55 activation may present an antinociceptive effect. Systemic administration of CID16020046, a GPR55 agonist, alleviated neuropathic pain behaviors and neuroinflammation in the spinal DH, where GPR55 expression was downregulated [150,151]. These opposing findings suggest that GPR55 may have distinct functions depending on its location, whether in the brain or the spinal cord.

##### Development of Agents Enhancing Endogenous Cannabinoid Signaling: Indirect Agonist Strategies

This therapeutic strategy is based on the pharmacological modulation of endogenous mechanisms involved in the synthesis, transport, and degradation of eCBs. By targeting these pathways, it is possible to amplify and prolong the physiological actions of eCBs without directly activating cannabinoid receptors. Indirect agonists encompass inhibitors of degradative enzymes such as FAAH and MAGL, blockers of endocannabinoid transport, and allosteric modulators that enhance the activity of endogenous ligands [84]. Collectively, these approaches offer a promising avenue for developing novel analgesics and anti-inflammatory agents with improved safety profiles compared to direct cannabinoid receptor agonists. However, this strategy has notable limitations. The inherently weak and transient antinociceptive effects of eCBs result from their rapid degradation and short half-life.

Among emerging therapeutic strategies, spinal modulation of MAGL has shown promise in alleviating neuropathic pain. MAGL represents the primary catabolic enzyme responsible for degrading 2-AG, so inhibiting it elevates endogenous 2-AG levels, enhancing CB2R binding and producing robust analgesic and anti-inflammatory effects. Systemic administration of MAGL inhibitors, including JZL184, MJN110, and KML29, effectively attenuates pain behaviors across diverse neuropathic pain models [152,153,154]. Intrathecal administration of JZL184 in inflammatory pain models also decreases nociceptive neurotransmission and central sensitization processes [155].

##### Development of Targeted Therapeutics for Selective Modulation of ECS Signaling Networks

Multiple signaling pathways within the ECS contribute to the diversity and selectivity of cannabinoid receptor-mediated responses (see Section 3.2).

- Allosteric modulators of cannabinoid receptors

Allosteric modulators offer a significant therapeutic advantage because they can selectively alter receptor activity, reducing the risk of receptor desensitization and drug tolerance [117]. Examples of allosteric modulators of the ECS include cannabidiol and pregnenolone. Cannabidiol acts as a negative allosteric modulator of the CB1R, which reduces the psychoactive effects of THC. It also modulates CB2R and selectively affects the activation of specific G protein subunits linked to these receptors. Pregnenolone, an endogenous steroid, also functions as a negative allosteric modulator of CB1R. It is released in response to high CB1R activation (e.g., after THC use) and acts as a protective mechanism against receptor overstimulation [156]. Similarly, positive allosteric modulation of TRPV1 can induce selective, lasting inactivation in neuronal elements, offering a promising approach for pain treatment [157].

- Drug design for cannabinoid receptor dimer

Compounds that selectively target cannabinoid receptor dimers represent a paradigm shift in drug design. This approach controls their distinct pharmacological properties, which differ from those of their monomeric forms. Heterodimeric cannabinoid receptors, similar to opioid heterodimers, may regulate and protect the nervous system during chronic antinociceptive modulation. This could help to: (1) prevent complete desensitization or tolerance to opioids, (2) provide more balanced pain signaling, and (3) contribute to emotional balance by reducing anxiety and depression [158].

Bivalent ligands stand out as another key innovation. These agents consist of two drug-like molecules (pharmacophores) linked by a flexible linker. This unique design enables them to simultaneously bind to both sites on a receptor dimer, providing high selectivity and potency for these specific complexes without affecting individual receptor monomers. For example, bivalent ligands targeting specific dimers, such as CB1R-OX1 (orexin), CB1R-DOR (delta opioid), and CB1R-5HT2A (serotonin), have confirmed superior affinity and selective effects in cells co-expressing both receptors, compared to either monomer alone [159,160,161].

- Biased agonists in cannabinoid pharmacology

Biased agonists, also known as functionally selective ligands, represent an advanced pharmacological strategy that capitalizes on GPCRs’ intrinsic ability to adopt multiple active conformations. Each conformation can selectively engage distinct intracellular signaling pathways, thereby enabling more precise therapeutic effects [162].

In the ECS, biased agonism is pertinent to both CB1R and CB2R. These GPCRs can trigger distinct intracellular signaling pathways depending on the specific agonist. This functional selectivity opens the door to the development of drugs with optimized pharmacodynamic profiles. For example, a biased agonist could be created to activate a G protein-dependent pathway for pain relief while avoiding a beta-arrestin-dependent pathway that might cause adverse effects. To date, biased agonists targeting CB1R and especially CB2R, such as LY2828360 and CB-05, have demonstrated efficacy in preclinical models of neuropathic pain [122,163,164].

#### 4.2.2. Other Strategies

##### Immune Response Modulation

Modulation of the immune response is a crucial strategy in managing neuropathic pain. One of the most promising approaches involves using inhibitors that target the activation of spinal glial cells, particularly microglia and astrocytes, which play a central role in neuroinflammation and pain sensitization. However, glial cells are also essential for maintaining homeostasis of the central nervous system. They perform crucial physiological functions such as phagocytosis, regulation of synaptic plasticity, and protection against pathogens. Therefore, therapeutic interventions must be carefully designed to suppress pathological glial activity while preserving their beneficial roles selectively [130,165].

Contemporary and emerging therapeutic strategies aimed at neuroinflammatory mechanisms in neuropathic pain can be broadly classified into four main categories: (1) selective inhibitors of microglial activation, (2) modulators of pro-inflammatory signaling pathways, (3) regulators of pathological astrocytic activity, and (4) pharmacological agents that interfere with aberrant intercellular glial communication.

- Selective inhibitors of microglial activation

Sustained microglial activation in the spinal cord following peripheral nerve injury perpetuates a neuroinflammatory cascade that enhances nociceptive transmission and facilitates central sensitization (see “Microglia-dependent pronociceptive mechanisms”). Therapeutic interventions targeting microglial activation interrupt this neuroinflammatory amplification loop, attenuate the release of pronociceptive mediators, and restore homeostatic spinal inhibitory modulation [166].

Among pharmacological modulators of microglial function, minocycline, a second-generation tetracycline antibiotic, has emerged as a widely studied therapeutic candidate. Preclinical studies show that prophylactic minocycline administration prevents the development of mechanical hyperalgesia, tactile allodynia, and pathological changes in glial glutamate transport. However, its clinical translation has produced mixed results, with significant efficacy observed for conditions such as chemotherapy-induced peripheral neuropathy and diabetic neuropathy but inconsistent effects for lumbar radiculopathy and post-surgical neuropathy following carpal tunnel release [167].

Other therapeutic interventions targeting microglial activation have also been evaluated. Intrathecal administration of p38 MAPK inhibitors such as SB203580 or FR167653 at early and late stages of nerve injury effectively reduces neuropathic pain [168,169]. Similarly, pentoxifylline, a non-selective phosphodiesterase inhibitor and TNF antagonist, alleviates mechanical allodynia in neuropathic pain [69]. Another promising approach involves antagonizing CGRP, which can modulate the p38-NF-kappa-B pathway in microglia [170].

A particularly promising strategy is targeting the colony-stimulating factor 1 receptor with selective inhibitors, such as PLX5622, as this receptor is crucial for microglia survival. Its inhibition can either deplete microglia or reprogram them into a neuroprotective state [130,131].

Additionally, cannabinoid receptor agonists inhibit microglial migration and proliferation (see above). Another advanced strategy focuses on promoting the anti-inflammatory M2 microglial phenotype using adenosine A3 receptor agonists or modulating signaling pathways of anti-inflammatory cytokines such as IL-10 and TGF-beta [171,172].

- Modulators of pro-inflammatory signaling pathways

Modulating spinal glial inflammatory signaling is a fundamental therapeutic strategy in conditions where glial cells amplify nociceptive responses following nerve injury. A central target in this context is the NLRP3 inflammasome, a multiprotein complex that orchestrates the maturation and release of pro-inflammatory cytokines such as IL-1 beta and IL-18 [173,174]. The use of selective NLRP3 inflammasome inhibitors, like MCC950, represents an innovative therapeutic approach. These agents effectively block both canonical and non-canonical inflammasome activation [173]. Furthermore, antagonism of IL-1 beta with neutralizing antibodies or the IL-1 receptor antagonist (anakinra) has shown promising results in both experimental and preliminary clinical studies [175]. 

Other therapeutic strategies include the use of complement inhibitors, which prevent microglial activation and localized inflammation, and non-steroidal anti-inflammatory drugs, which contribute to microglial deactivation and reduce neuroinflammation by inhibiting cyclooxygenase (COX), prostaglandin E2 synthesis, and activating PPAR-gamma.

- Regulators of pathological astrocytic activity

Regulating astrocyte activation and restoring their homeostatic function, including regulating extracellular glutamate and maintaining the blood–brain barrier, is crucial for preventing the amplification of nociceptive signaling (see “Astrocyte-dependent pronociceptive mechanisms”). Key intracellular glial signaling pathways, such as JAK/STAT, p38 MAPK, and NF-kappa-B, play central roles in driving astrocytes toward a pro-inflammatory reactive phenotype. Experimental models demonstrate that inhibition of the JAK2/STAT3 pathway with agents like AG490 reduces GFAP expression and pro-inflammatory cytokine production. This molecular effect leads to a significant decrease in mechanical and thermal allodynia [176]. Similarly, blocking p38 MAPK with compounds such as SB203580 restores glutamate uptake via GLT-1 and GLAST transporters, effectively limiting excitotoxicity and central sensitization [177]. Additionally, modulation of NF-kappa-B signaling with I-kappa-B kinase inhibitors, such as BMS-345541, decreases pro-inflammatory gene transcription in astrocytes and facilitates recovery of the blood–brain barrier [178].

- Pharmacological agents that interfere with aberrant intercellular glial communication.

Bidirectional communication between neurons and glial cells is critical for the development of central sensitization. Damaged neurons release molecular patterns that activate toll-like receptors on glial cells [179]. In turn, activated glial cells secrete pro-inflammatory mediators that modulate neuronal excitability and promote maladaptive synaptic plasticity [130]. Therefore, interventions targeting this neuron-glial intercellular communication may provide novel therapeutic avenues for managing neuropathic pain and restoring neuronal microenvironment homeostasis. Examples of such strategies include connexin-43 hemichannel inhibitors, which are crucial for the pathological release of ATP and glutamate from astrocytes. Compounds such as carbenoxolone have demonstrated efficacy in reducing gliotransmitter release [180]. More specific agents, such as the connexin-43 mimetic peptides (Gap26 and Gap27), offer a distinct therapeutic advantage by selectively inhibiting hemichannels while sparing the physiological functions of gap junctions. This selectivity helps preserve astrocytic homeostasis and intercellular communication, making them promising candidates for precise modulation of glial activity in chronic pain conditions [181]. Similarly, the fractalkine/CX3CR1 pathway represents a critical therapeutic target in neuron-microglia communication. Neuronal fractalkine binds to microglial CX3CR1 receptors, activating downstream signaling cascades including p38 MAPK. Neutralizing antibodies or selective CX3CR1 antagonists have demonstrated significant analgesic efficacy by reducing microglial activation [182].

##### Endocrine Response Modulation

Estrogens, progesterone, and other neurosteroids have shown neuroprotective and antinociceptive effects in spinal cord injury models by reducing inflammation and preventing neuronal death. Progesterone has emerged as an essential modulator due to its capacity to regulate inflammatory processes through classical progesterone receptors (PR), which act as ligand-dependent transcription factors in neurons and glial cells [183]. PR plays a decisive role in regulating cytokine production in endothelial cells and in inhibiting NF-kappa-B transactivation in the spinal cord [184]. Recent studies have underscored the importance of functional PR activity in modulating progesterone’s effects on cytokine expression following spinal cord injury, emphasizing its potential role in regulating neuroinflammation in neuropathic pain [185]. Collectively, these findings position progesterone-mediated signaling pathways as promising targets for the development of more effective therapies for spinal neuropathic pain.

##### Pharmacogenomics

Applying pharmacogenomics to neuropathic pain treatment holds significant promise for optimizing therapeutic outcomes by enabling drug and dose selection tailored to individual genetic profiles. Genetic variations in drug-metabolizing enzymes such as CYP2D6 affect the biotransformation of opioids and cannabinoids, influencing systemic exposure and the risk of adverse effects [186].

Moreover, polymorphisms in genes encoding receptors and signaling molecules involved in nociceptive transmission, such as single-nucleotide polymorphisms (SNPs) in *OPRM1* (mu-opioid receptor), *CNR1/CNR2* (CB1R/CB2R cannabinoid receptors), and *SCN9A* (Nav1.7 sodium channel), can modulate analgesic efficacy and pharmacological tolerance. Consequently, these variations predispose patients to variable therapeutic outcomes and toxicity profiles [187]. Association studies have further identified *IL1B* and *TNF* haplotypes that make individuals highly vulnerable to neuroinflammatory responses and suboptimal outcomes with glial-targeted anti-inflammatory therapies. These findings support the selective use of agents such as p38 MAPK inhibitors or IL-1 beta antagonists in patients with a genetic predisposition [188].

The integration of genomic panels specific to neuropathic pain into clinical practice could reduce reliance on empirical drug trials, minimize adverse effects and enhance therapeutic precision.

## 5. Conclusions

The spinal ECS is a key modulator of nociceptive signaling, with a dual function that depends on whether it is operating in a healthy or a pathological context. Under normal conditions, ECS activation inhibits nociceptive transmission through both presynaptic and postsynaptic mechanisms, producing a protective antinociceptive effect. However, following persistent nerve injury or chronic inflammation, as seen in neuropathic pain, the ECS can become dysfunctional or excessively overactivated, paradoxically contributing to central sensitization and the persistence of pain.

This functional ambivalence is determined by multiple interconnected factors, including anatomical location and receptor density, glial activation state, the local neuroimmune microenvironment, the duration and intensity of nociceptive stimuli, and interactions with other endogenous modulatory pathways. Understanding these mechanisms is essential to understanding when the ECS acts as an antinociceptive system and when it becomes a facilitator of hypersensitivity and pathological neural plasticity.

From a therapeutic perspective, ECS duality represents both a challenge and an opportunity. Pharmacological manipulation of the ECS, through selective CB1R and CB2R agonists, FAAH and MAGL enzyme inhibitors, allosteric modulators, or combined strategies including glial modulators, constitutes a promising avenue for developing innovative treatments targeting neuropathic pain. However, the success of these interventions critically depends on a precise understanding of the pathophysiological context of eCB pathways and the evolutionary stage of the pathology.

Despite preclinical advances, translational limitations persist due to interspecies differences and imperfect representation of human pain in animal models. Additionally, identification of cellular types responsible for cannabinoid effects and signaling profiles necessary for optimal clinical efficacy remains incomplete. Future research should focus on defining cannabinoid response phenotypes, developing predictive biomarkers and creating more sophisticated preclinical models that reflect the functional bidirectionality of the ECS. Only through such advances can we progress toward precision medicine that transforms the therapeutic potential of the ECS into sustainable clinical benefits for patients with chronic neuropathic pain.

In summary, elucidating and therapeutically targeting the functional duality of the spinal ECS represents not merely a pharmacological challenge but a paradigmatic opportunity to transform neuropathic pain management (see Table 2).

## Figures and Tables

**Table 1:** Therapeutic strategies for the management of neuropathic pain at the spinal level: general considerations.

Strategy	Mechanism/Target	Evidence	Examples/ Specific Agents	Advantages	Limitations/Notes
Intrathecal (IT) administration	Direct delivery into CSF, modulation at the dorsal horn and DRG	Robust preclinical/translational support	Cannabinoid agonists (levonantradol, WIN55,212-2, CP-55,940) FAAH inhibitors (URB597, AA-5-HT) MAGL inhibitors (URB602)	Lower doses, reduced systemic effects	Requires specialized technique, careful patient selection
Endocannabinoid (eCB) modulation	Modulation of endogenous eCB levels through regulation of synthesis/transport, selective activation of receptor subtypes, and intervention in supramolecular complexes	Enhances analgesia; context/time-dependent plasticity	Enzyme inhibitors (FAAH, MAGL), Selective CB2R agonists and Targeting of receptor heteromers	Potentiates endogenous signaling with fewer central effects Considers glial cell heterogeneity (M1/M2 microglia, A1/A2 astrocytes)	CB1R desensitization; variable responses depending on microenvironment Activation of CB1R on inhibitory interneurons in neuropathic pain can induce synaptic disinhibition by reducing the presynaptic release of inhibitory neurotransmitters
Glial-targeted therapies	Decrease microglial/astrocytic activation and neuroinflammation.	Strong preclinical support; mixed clinical results	See ‘Modulation of immune response’ in the specific strategies section.	Reduces central sensitization	Must preserve homeostatic glial functions
Multimodal analgesia	A combination of different analgesic therapies to simultaneously target multiple pain pathways	Synergistic, less tolerance	See Section 4.1.4 in the specific strategies section	Dose sparing and reduced adverse events	Requires rational design of combinations and timing

**Table 2:** Therapeutic strategies for the management of neuropathic pain at the spinal level: specific considerations of endocannabinoid system (ECS)-dependent strategies.

		Intervention	Molecular Focus	Spinal Mechanism	Examples	Advantages	Limitations/Warnings
ECS-related strategies	Receptor-Based Intervention	CB1R agonism IT	Neuronal CB1R	Antinociception ↓ eEPSCs; ↓ HCN1/2 in DRG (↓ excitability)	ACEA, THC	Potent antihyperalgesic	CB1R desensitization/downregulation with sustained exposure
		CB2R agonism IT	Neuronal/glial CB2R	Antinociception/anti-inflammatory; ↓ central sensitization; low tolerance	HU-308, L-759,632, AM1710, AM724, THC derivatives, Sch35966	Safer profile (minimal CNS adverse effects)	Glia-context variability requires high selectivity
		Non-CB1/CB2 receptors IT	Neuronal/glial TRPV1 channel, PPARs and GPR119 receptor	TRPV1 silencing/antagonism; PPARs regulate glial activity and directly contribute at the spinal level to decrease hyperexcitability and prevent excessive propagation of nociceptive signals; The GPR119 receptor modulates ascending pain transmission at the spinal level through distinct G-protein-coupling mechanisms	Selective antagonists, negative allosteric modulators, inhibition of phosphorylation cascades, promotion of TRPV1 internalization and reduction of glial neuroinflammation (example: TRPV1-targeting siRNA); Pioglitazone, a PPARγ agonist; AS1269574, a GPR119 agonist)	Targeted effects; potential synergy with ECS Some antinociceptive effects of PPARγ agonists (such as pioglitazone) in neuropathic pain models are extremely rapid (within 5 minutes after intrathecal administration), indicating a non-genomic mechanism in addition to the classical nuclear pathway	TRPV1: thermoregulation issues; PPAR agonist efficacy exhibits significant context-dependent variability Although AS1269574 suggests a role for GPR119 in spinal neuropathic pain, prior evidence lacks pharmacological specificity
	Selective Modulation of Endocannabinoid System Signaling Networks	Metabolic enzymes of eCBs Endocannabinoid transport systems	Neuronal/glial degradation enzymes (FAAH, MAGL), membrane transporters (EMT/AMT) and synthesis enzymes (NAPE-PLD, DAGL)	↑ AEA/2-AG; potentiates CB2R/CB1R	URB597 (FAAH), URB602 (MAGL), JZ	Reduced psychoactive liability compared to direct agonists, with sustained analgesic efficacy and diminished tolerance development, particularly when combined with glial modulators	Sometimes, weaker/transient effects, limited specificity
		Heteromers/dimers	Neuronal/glial CB1–OX1/CB1–DOR/CB1–5HT2A	Distinct pharmacology, bivalent ligands are highly selective	Bivalent ligands	Higher potency/selectivity	Early stage; limited clinical translation
		Biased agonism	Neuronal/glial CB1R/CB2R	Favor G-protein vs β-arrestin pathways for analgesia	LY2828360 (G protein-biased CB2R agonist), CB-05 (G protein-biased CB2R agonist)	Optimizes efficacy/adverse effects	No clinical consensus yet; requires pathway biomarkers
Other Strategies	Glial and immune modulation (beyond ECS)	Microglial inhibitors	Microglia (M1 → M2; survival/activation)	↓ p38 MAPK; ↓ cytokines; possible depletion/reprogramming	Minocycline; p38i (SB203580, FR167653); CSF1R i (PLX5622); pentoxifylline; A3 agonists; IL-10/TGF-β pathways	These compounds suppress microglial activation, attenuate proinflammatory cytokine signaling cascades and restore homeostatic inhibitory modulation within the spinal cord	Must preserve homeostatic functions; therapeutic window critical
		Proinflammatory pathways	Inflammasome/IL-1β/COX	NLRP3 inhibition; anti-IL-1β; ↓ PGE2; ↑ PPAR-γ	MCC950; anakinra/anti-IL-1β; NSAIDs	Defined effects; combinable	Systemic immunosuppression risk (mitigated with local delivery)
		Astrocytic regulation	Astrocyte JAK/STAT, p38-MAPK, NF-κB; GLT-1/GLAST	↓ GFAP/cytokines; restores glutamate uptake; BSCB effects	AG490 (JAK2/STAT3); SB203580; IKKi (BMS-345541)	Reduces nociceptive amplification	Timing/dose critical to avoid homeostatic disruption
		Neuro-glial communication	Connexin-43; fractalkine/CX3CR1; CGRP-p38-NF-κB	↓ pathological ATP/glutamate release; ↓ microglial activation	Carbenoxolone; Cx43 peptides (Gap26/Gap27); anti-CX3CR1	Interrupts sensitization loops	Specificity and local delivery are key
	Endocrine response modulation	Neurosteroids	Neuronal/glial PR	Anti-inflammatory; NF-κB inhibition; cytokine regulation	Progesterone	Neuroprotective; reduces persistent neuroinflammation	PR-dependency; timing and dosing critical
	Pharmacogenomics	Detect Individual genetic variations in genes encoding metabolizing enzymes, transporters, and receptors to determine personalized drug responses	Genetic variations in drug-metabolizing enzymes—such as CYP2D6 and CYP3A4; Polymorphisms in nociceptive genes—such as SNPs in OPRM1 (μ-opioid receptor), CNR1/CNR2 genes (encoding CB1R/CB2R) and SCN9A (Nav1.7 sodium channel), IL1B and TNF haplotypes predisposing to heightened neuroinflammation and poor anti-inflammatory therapy outcomes	Optimize spinal analgesic interventions.		Personalized therapy; reduces trial-and-error	Requires validated panels and access to testing
Multimodal analgesia		A combination of different analgesic therapies to simultaneously target multiple pain pathways	Neuronal/glial	Synergistic antinociceptive effects of cannabinoid and other receptors in neuronal and glial cells → Crosstalk with complementary antinociceptive systems	p38 MAP kinase inhibitors + opioid agonists; Cannabinoids + opioids; FAAH and MAGL inhibitors + glial modulators; Minocycline + propentofylline (for microglial and astrocyte inhibition)	Co-expression targets in dorsal horn; synergy → Dose reduction Reduced opioid tolerance/adverse effects Break central sensitization	Timing and immune monitoring

## Data Availability

Not applicable.

## Abbreviations

**Abbreviation**	**Name**	**Category**
2-AG	2-Arachidonoylglycerol	Endocannabinoid (Neurotransmitter)
5-HT	5-Hydroxytryptamine	Neurotransmitter
5HT2A	5-Hydroxytryptamine receptor 2A	Receptor (GPCR)
AA	Arachidonic Acid	Signal Molecule
AA-5-HT	N-Arachidonoyl Serotonin	Signal Molecule/Endocannabinoid
A-beta fibers	Large-diameter myelinated sensory fibers	Cell/Structure
A-delta fibers	Small-diameter thinly myelinated sensory fibers	Cell/Structure
AEA	Anandamide	Endocannabinoid (Neurotransmitter)
Ado	Adenosine	Neurotransmitter
AMPA	Alpha-amino-3-hydroxy-5-methyl-4-isoxazolepropionic acid	Receptor (Ionotropic)
AMPAR	Alpha-amino-3-hydroxy-5-methyl-4-isoxazolepropionic acid receptor (glutamate receptor)	Receptor (Ionotropic)
AP-1	Activator Protein-1	Transcription Factor
ASIC	Acid-Sensing Ion Channel	Ion Channel
BDNF	Brain-Derived Neurotrophic Factor	Neurotrophin/Signal Molecule
BB	Blood–Brain Barrier	Structure
BSCB	Blood–Spinal Cord Barrier	Structure
βarr2	Beta-Arrestin 2	Signal Molecule/Protein
Ca^2+^	Calcium ion	Ion
CaM	Calmodulin	Signal Protein
CAV	Voltage-Gated Calcium Channel	Ion Channel
CB	Cannabinoid Receptor (generic)	Receptor (GPCR)
CB1R	Cannabinoid Receptor Type 1	Receptor (GPCR)
CB2R	Cannabinoid Receptor Type 2	Receptor (GPCR)
CB1/2	Cannabinoid Receptor Types 1 and 2	Receptor (GPCR)
CCL2	CC Chemokine Ligand 2	Chemokine (Signal)
CCR2	CC Chemokine Receptor 2	Receptor (GPCR)
cAMP	Cyclic Adenosine Monophosphate	Second Messenger
C-jun	Jun Proto-Oncogene	Transcription Factor
C-myc	Myc Proto-Oncogene	Transcription Factor
CGRP	Calcitonin Gene-Related Peptide	Neuropeptide/Signal Molecule
CNR1/CNR2	CB1R/CB2R genes	Gene
CNS	Central Nervous System	Structure
COX/COX-2	Cyclooxygenase/Cyclooxygenase-2	Enzyme
CREB	cAMP Response Element-Binding Protein	Transcription Factor
CSF	Cerebrospinal Fluid	Structure/Fluid
CSF1R	Colony-Stimulating Factor 1 Receptor	Receptor (Tyrosine Kinase)
Cx43	Connexin-43	Gap Junction Protein
CX3CL1	CX3C Chemokine Ligand 1	Chemokine (Signal)
CX3CR1	CX3C Chemokine Receptor 1	Receptor (GPCR)
CYP2D6/CYP3A4	Cytochrome P450	Enzyme
DAG	Diacylglycerol	Signal/Lipid
DAGL/DAGLα	Diacylglycerol Lipase (α isoform)	Enzyme
DOR	Delta Opioid Receptor	Receptor (GPCR)
DRG	Dorsal Root Ganglion	Structure/Cell Group
eCB/eCBs/ECBs	Endocannabinoids	Neurotransmitter
ECS	Endocannabinoid System	Signaling System
eEPSCs	Evoked Excitatory Postsynaptic Currents	Electrophysiological Event
EMT/AMT	Endocannabinoid/Anandamide Membrane Transporter	Transporter
ERK/ERK1/2	Extracellular Signal-Regulated Kinase(s)	Signal Protein/Enzyme
erbB2/3/4	Erythroblastic Leukemia Viral Oncogene Homolog	Receptor (Tyrosine Kinase)
FAAH/FAAH-1/FAAH-2	Fatty Acid Amide Hydrolase	Enzyme
GABA	Gamma-Aminobutyric Acid	Neurotransmitter
GABAR	GABA Receptor	Receptor (Ionotropic/Metabotropic)
Gαi/o	G Protein αi/o Subunit	Signal Molecule/Protein
GDR	Dorsal Root Ganglion	Structure/Cell Group
GFAP	Glial Fibrillary Acidic Protein	Glial Cell Marker
GIRK	G Protein-Coupled Inwardly Rectifying Potassium Channel	Ion Channel
Glu	Glutamate	Neurotransmitter
GLT-1/GLAST	Astrocytic Glutamate Transporters	Transporter
Gly	Glycine	Neurotransmitter
GlyR	Glycine Receptor	Receptor (Ionotropic)
GPR55	G Protein-Coupled Receptor 55	Receptor (GPCR)
GPR119	G Protein-Coupled Receptor 119	Receptor (GPCR)
HCN/HCN1/2	Hyperpolarization-Activated Cyclic Nucleotide-Gated Channel	Ion Channel
ICAM	Intercellular Adhesion Molecule	Adhesion Molecule
IL	Interleukin	Cytokine/Signal
IL-1 beta	Interleukin 1 beta	Cytokine
IL-1R	Interleukin 1 receptor	Receptor (Cytokine)
IL-6	Interleukin 6	Cytokine
IL-10	Interleukin 10	Cytokine
IL-33	Interleukin 33	Cytokine
iNOS	Inducible Nitric Oxide Synthase	Enzyme
IP_3_	Inositol Trisphosphate	Second Messenger
IT	Intrathecal	Route of Administration/Procedure
JAK/STAT	Janus Kinase/Signal Transducer and Activator of Transcription	Signal Protein/Pathway
JNK	c-Jun N-terminal Kinase	Signal Protein/Enzyme
K^+^	Potassium Ion	Ion
KATP	ATP-Sensitive Potassium Channel	Ion Channel
KCC2	Potassium-Chloride Cotransporter 2	Transporter
Kv4.2	Potassium Voltage-Gated Channel Subfamily D Member 2	Ion Channel
KROX 24	Transcription Factor KROX 24	Transcription Factor
LFA-1	Lymphocyte Function-Associated Antigen 1	Adhesion Molecule
MAGL	Monoacylglycerol Lipase	Enzyme
MAPK	Mitogen-Activated Protein Kinase	Signal Protein/Enzyme
M1/M2	Microglial Phenotypes	Cell/Phenotype
MMP-2/MMP-9	Matrix Metalloproteinases	Enzyme
MeCP2	Methyl CpG-Binding Protein 2	Epigenetic Marker/Protein
mCB1R	Mitochondrial Cannabinoid Receptor 1	Receptor (GPCR)
Na^+^	Sodium Ion	Ion
NAPE-PLD	N-Acyl Phosphatidylethanolamine Phospholipase D	Enzyme
Nav1.7/SCN9A	Voltage-Gated Sodium Channel/Gene	Ion Channel/Gene
NFAT	Nuclear Factor of Activated T-Cells	Transcription Factor
NF-kappa-B	Nuclear Factor Kappa B	Transcription Factor
NGF	Nerve Growth Factor	Neurotrophin/Signal Molecule
NK1/NK2	Neurokinin 1/2 Receptor	Receptor (GPCR)
NMDA/NMDAR	N-Methyl-D-Aspartate Receptor	Receptor (Ionotropic)
NO	Nitric Oxide	Signal Molecule
NSAIDs	Non-Steroidal Anti-Inflammatory Drugs	Drug Class
OPRM1	Mu-Opioid Receptor Gene	Gene
OPCs	Oligodendrocyte Precursor Cells	Cell Type
OX1	Orexin receptor type 1	Receptor (GPCR)
p38/P-p38	Stress-Activated MAP Kinases	Signal Protein/Enzyme
PARP	Poly(ADP-ribose) Polymerase	Enzyme
PBMC	Peripheral Blood Mononuclear Cell	Cell Type
P-CREB	Phosphorylated cAMP Response Element-Binding Protein	Transcription Factor (activated)
P-ERK	Phosphorylated ERK	Signal Protein (activated)
PECAM	Platelet Endothelial Cell Adhesion Molecule	Adhesion Molecule
PG/PGE_2_	Prostaglandin/Prostaglandin E_2_	Signal Molecule
PIP_2_	Phosphatidylinositol 4,5-Bisphosphate	Second Messenger/Lipid
PKC	Protein Kinase C	Enzyme
PKA	Protein Kinase A	Enzyme
PLC	Phospholipase C	Enzyme
PPAR/PPARs	Peroxisome Proliferator-Activated Receptor(s)	Nuclear Receptor/Transcription Factor
PR	Progesterone Receptor	Nuclear Receptor
ROCK	Rho-Associated Protein Kinase	Enzyme
RhoA	Ras Homolog Family Member A	Signal Molecule/Protein
RE	Reticulum Endoplasmic	Organelle
SCN9A	Sodium Channel, Voltage-Gated, Type IX, Alpha Subunit Gene	Gene
siRNA	Small Interfering RNA	Genetic Tool
SNPs	Single Nucleotide Polymorphisms	Genetic Variation
STAT/STAT3	Signal Transducer and Activator of Transcription	Signal Protein/Transcription Factor
TGF-beta	Transforming Growth Factor Beta	Cytokine/Signal
T-cells	T- lymphocytes	Cell Type
TNF	Tumor Necrosis Factor Alpha	Cytokine
TNFR	Tumor Necrosis Factor Receptor	Receptor
TRP	Transient Receptor Potential Channel Family	Ion Channel
TRPA1	Transient Receptor Potential Ankyrin 1	Ion Channel
TRPM3	Transient Receptor Potential Melastatin 3	Ion Channel
TRPV1	Transient Receptor Potential Vanilloid 1	Ion Channel
VGCC	Voltage-Gated Calcium Channel	Ion Channel
